# In silico-guided metabolic engineering of *Bacillus subtilis* for efficient biosynthesis of purine nucleosides by blocking the key backflow nodes

**DOI:** 10.1186/s13068-022-02179-x

**Published:** 2022-08-11

**Authors:** Aihua Deng, Qidi Qiu, Qinyun Sun, Zhenxiang Chen, Junyue Wang, Yu Zhang, Shuwen Liu, Tingyi Wen

**Affiliations:** 1 CAS Key Laboratory of Pathogenic Microbiology and Immunology, Institute of Microbiology, Chinese Academy of Sciences, Beijing, 100101 China; 2 College of Life Sciences, University of Chinese Academy of Sciences, Beijing, 100049 China; 3 Savaid Medical School, University of Chinese Academy of Sciences, Beijing, 100049 China; 4 China Innovation Academy for Green Manufacture, Chinese Academy of Sciences, Beijing, 100049 China

**Keywords:** *Bacillus subtilis*, Genome-scale metabolic network model, Metabolic engineering, Backflow node, Purine nucleosides, Chassis bacterium

## Abstract

**Background:**

Purine nucleosides play essential roles in cellular physiological processes and have a wide range of applications in the fields of antitumor/antiviral drugs and food. However, microbial overproduction of purine nucleosides by de novo metabolic engineering remains a great challenge due to their strict and complex regulatory machinery involved in biosynthetic pathways.

**Results:**

In this study, we designed an in silico-guided strategy for overproducing purine nucleosides based on a genome-scale metabolic network model in *Bacillus subtilis*. The metabolic flux was analyzed to predict two key backflow nodes, Drm (purine nucleotides toward PPP) and YwjH (PPP–EMP), to resolve the competitive relationship between biomass and purine nucleotide synthesis. In terms of the purine synthesis pathway, the first backflow node Drm was inactivated to block the degradation of purine nucleotides, which greatly increased the inosine production to 13.98–14.47 g/L without affecting cell growth. Furthermore, releasing feedback inhibition of the purine operon by promoter replacement enhanced the accumulation of purine nucleotides. In terms of the central carbon metabolic pathways, the deletion of the second backflow node YwjH and overexpression of Zwf were combined to increase inosine production to 22.01 ± 1.18 g/L by enhancing the metabolic flow of PPP. By switching on the flux node of the glucose-6-phosphate to PPP or EMP, the final inosine engineered strain produced up to 25.81 ± 1.23 g/L inosine by a *pgi*-based metabolic switch with a yield of 0.126 mol/mol glucose, a productivity of 0.358 g/L/h and a synthesis rate of 0.088 mmol/gDW/h, representing the highest yield in de novo engineered inosine bacteria. Under the guidance of this in silico*-*designed strategy, a general chassis bacterium was generated, for the first time, to efficiently synthesize inosine, adenosine, guanosine, IMP and GMP, which provides sufficient precursors for the synthesis of various purine intermediates.

**Conclusions:**

Our study reveals that in silico-guided metabolic engineering successfully optimized the purine synthesis pathway by exploring efficient targets, which could be applied as a superior strategy for efficient biosynthesis of biotechnological products.

**Supplementary Information:**

The online version contains supplementary material available at 10.1186/s13068-022-02179-x.

## Background

As essential metabolites in organisms, purine intermediates participate in the metabolic synthesis of nucleic acids, energy and amino acids [1, 2]. Among them, purine nucleosides consist of adenosine, guanosine, inosine and xanthosine, which contain nucleosides and bases [3]. They have a wide range of applications in the fields of food and medicine as commercially important flavor enhancers and precursors of antitumor/antiviral drugs [4, 5]. Currently, tumors and influenza viruses seriously threaten the health of humans, triggering increasing demands for nucleoside products worldwide. Microbial fermentation is one of the major approaches for producing purine nucleosides, and investigations have been carried out to construct genetically engineered bacteria by metabolic engineering [4, 6–9]. It has been revealed that purine nucleosides are de novo synthesized via the glycolysis pathway (EMP, Embden–Meyerhof–Parnas pathway), pentose phosphate pathway (PPP), and purine synthesis pathway (Additional file 1: Figure S1). First, glucose-6-phosphate, the intermediate product synthesized by the first step of EMP, is catalyzed by the 5-step enzymatic reactions of PPP to form phosphoribosyl pyrophosphate (PRPP). Then, PRPP is catalyzed to form inosine monophosphate (IMP) through 11 steps of enzymatic reactions in the purine synthesis pathway. Finally, the precursor IMP is catalyzed to form various purine nucleotides, nucleosides, and nucleobases through the terminal purine synthesis pathway. Meanwhile, nucleobases can be directly catalyzed by the phosphoribosyltransferases to form nucleotides in the salvage pathway (Additional file 1: Figure S1). Thus, the synthesis of purine nucleosides requires many precursors and energy, which are difficult to accumulate in the cell because of strict and complex regulation [3, 10].

*Bacillus* is known to be superior for the fermentation of nucleosides with advantages of a safe producer, few byproducts, and a simple separation process [11, 12]. In *B. subtilis* 168, inosine titer was increased to 6 g/L by disrupting the conversion of IMP to AMP and XMP, the degradation of inosine, and the repressor PurR and 5’-UTR of the *pur* operon [13]. Furthermore, nucleoside efflux transporters have been reported to increase the production of extracellular purine nucleosides [9]. Under guidance of transcriptional and metabolite pool analysis, overexpression of *prs*, *purF* and *purA* in *B. subtilis* XGL has been reported to increase PRPP concentration and *pur* operon transcription, which achieved adenosine accumulation to 7.04 g/L [7]. In 2019, Li et al. inactivated the GMP synthetase gene *guaA* and optimized the growth condition to increase adenosine titer from 7.40 to 14.39 g/L in *B. subtilis* A509 [6]. Besides, *Escherichia coli* is another industrial strain that is applied to nucleoside synthesis and has been reported to produce 6.7–7.5 g/L inosine by overexpressing the *prs* and *purF* genes with inactivation of the *purA*, *deoD*, *purF*, *purR*, *add*, *edd*, *pgi* and *xapA* genes [14]. Our previous study has shown that a de novo engineered strain with a clear genetic background was constructed to accumulate 7.6 g/L inosine by deleting the *purA* and *deoD* genes in *B. subtilis* W168 [11]. The flux distribution of the whole cell revealed that purine synthesis has a long metabolic pathway and its carbon flux is carried by PPP, which is not the main intracellular carbon metabolism pathway. Furthermore, nucleoside synthesis is strictly and complexly regulated in the cell, remaining a great challenge for the overproduction of nucleosides by de novo metabolic engineering [15, 16]. Overall, previous efforts to construct engineered strains mostly focused on the metabolic pathways directly associated with purine nucleoside biosynthesis, which often resulted in slow growth and relatively low production of desired metabolites [11, 17]. Therefore, it is necessary to adopt a rationally designed engineering strategy to achieve the effective synthesis of purine nucleosides.

With the rapid development of whole-genome sequencing and multi-omics technologies, genome-scale metabolic network models (GEMs) have been developed by constructing metabolic networks with the aid of computer technology [18, 19]. Based on the principle of gene–protein–reaction relationships, GEM is constructed to link genes to the proteins that catalyze reactions in the network. A series of biochemical reactions in the cell are responsible for the synthesis of energy and metabolites. Thus, the constructed models can be widely used in quantitative predictions of cell growth and desired products under metabolically/environmentally disturbed conditions [20]. Until now, GEMs have been undergone multiple generations of upgrades to continuously improve the completeness of models, the accuracy of prediction and the scope of application in industrial microorganisms, such as *E. coli*, *B. subtilis*, *Corynebacterium glutamicum*, and *Saccharomyces cerevisiae* [18, 21–23]. Among them, the *i*Bsu1103V2 model for *B. subtilis* accounts for 1103 open reading frames and 1451 metabolic reactions involving 1156 metabolites. Compared with the available models in *B. subtilis*, it has been improved considerably at predictions of metabolites and viability of mutant strains in rich defined medium and glucose minimal media. Thus, the *i*Bsu1103V2 model provides an effective tool to conduct rational guidance for metabolic engineering and systems biology research in *B. subtilis* [24].

In this study, we take advantage of the *i*Bsu1103V2 model to analyze and predict the novel targets for increasing IMP flux without influencing biomass [25, 26]. An in silico-guided engineering strategy was designed to disrupt the backflow node (the pentose phosphate mutase Drm) of the purine synthesis pathway toward the PPP, release feedback inhibition of the purine operon, block the backflow node (the transaldolase YwjH) of the PPP to the EMP, and regulate the expression of key enzymes. Under the guidance of this rationally designed strategy, a universal chassis strain was de novo constructed to accumulate various purine intermediates. The final engineered strain has experimentally showed the highest yield of inosine in de novo engineered bacteria, which is of great significance for further understanding purine metabolite biosynthesis and its regulatory mechanism.

## Results

### Initial biosynthesis of purine nucleosides by the traditional engineering method

**Fig. 1:**
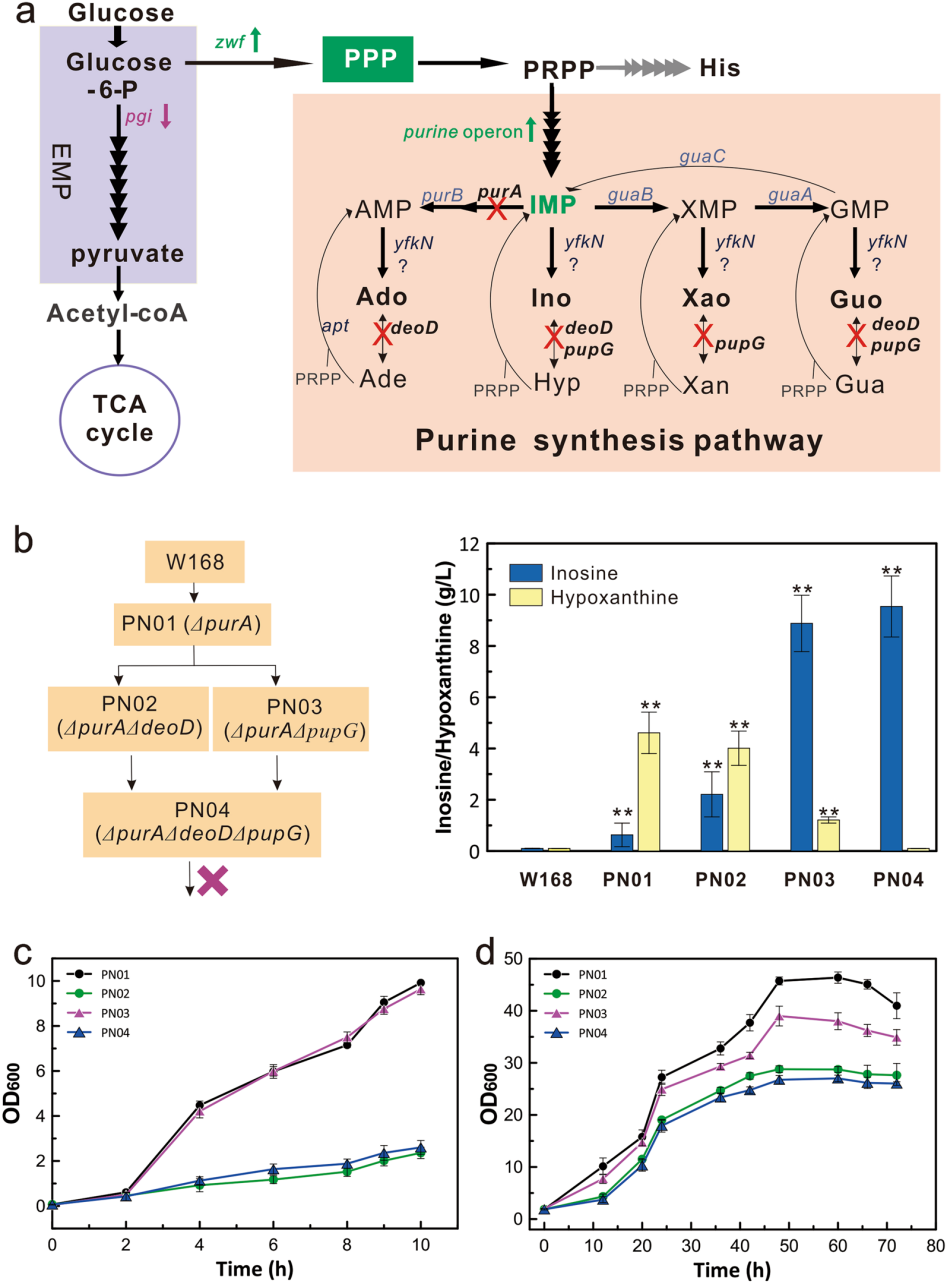
Traditional engineering of *Bacillus subtilis* for de novo synthesis of purine nucleosides. **a** Biosynthetic pathway of purine nucleosides in *B. subtilis*. *prs*, encoding PRPP synthetase; *purEKBCSQLFMNHD* (*pur* operon), encoding enzymes required to synthesize IMP from PRPP; *P*_*pur*_, purine operon promoter; *purA*, encoding adenylosuccinate synthase; *purB*, encoding adenylosuccinate lyase; *guaB*, encoding IMP dehydrogenase; *guaA*, encoding GMP synthetase; *guaC*, encoding GMP reductase; *deoD*, encoding purine nucleoside phosphorylase (PNP); *pupG*, encoding PNP; *apt*, encoding adenine phosphoribosyltransferase; *hpt*, encoding hypoxanthine–guanine phosphoribosyltransferase; *xpt*, encoding xanthine phosphoribosyltransferase; Ado, adenosine; Ino, inosine; Xao, xanthosine; Guo, guanosine; Ade, adenine; Hyp, hypoxanthine; Xan, xanthine; Gua, guanine. **b** Construction of engineered strains for de novo synthesis of inosine by traditional metabolic engineering. **c** The growth curve in seed cultivation. **d** Growth curve by shake-flask cultivation. Data shown are mean values from three biological replicates and a value of *P* less than 0.05 is regarded to be a significant difference with that of W168 strain using the T-test (**, *P* < 0.01)

IMP is an important precursor for synthesizing various purine nucleosides by 1–3 steps of catalytic reactions in the purine synthesis pathway of *B. subtilis*. Among various purine nucleosides, inosine is directly synthesized from IMP through a one-step reaction (Fig. 1a). To rapidly evaluate the engineering effect, inosine production was used as an indicator to determine the metabolic flow of purine nucleosides in this study. The *purA* gene encoding adenylosuccinate synthetase was first knocked out to block the adenosine synthesis branch and increased the inosine accumulation of the engineered strain PN01 (W168 *ΔpurA*). Then, the purine nucleoside phosphorylases (PNPs) DeoD and/or PupG, which are involved in the degradation of nucleosides, were inactivated to generate the engineered strains PN02 (W168 *ΔpurA ΔdeoD*), PN03 (W168 *ΔpurA ΔpupG*) and PN04 (W168 *ΔpurA ΔpupG ΔdeoD*) (Fig. 1b).

After shake-flask cultivation for 72 h, strain PN01 accumulated 0.62 ± 0.16 g/L inosine and 4.63 ± 0.22 g/L hypoxanthine formed by the degradation of inosine (Fig. 1b). Inosine titer increased in the *pupG*- and/or *deoD*-deficient strains PN02 (2.21 ± 0.88 g/L), PN03 (8.88 ± 1.10 g/L), and PN04 (9.54 ± 1.19 g/L) with improved yields (0.009 to 0.039 mol/mol glucose), productivities (0.009 to 0.133 g/L/h), and synthesis rates (0.002 to 0.038 mmol/gDW/h) (Additional File 2: Table S1). Moreover, the degradation of inosine (as shown by the hypoxanthine production) continuously decreased in these strains. These results suggested that *purA* inactivation resulted in a high concentration of hypoxanthine for inosine degradation, which was significantly reduced by deleting the *pupG* and *deoD* genes. However, cell growths of strains PN02, PN03 and PN04 were greatly decreased, especially slow for PN03 and PN04 in seed cultivations, which negatively affected the engineered strain's characteristics and was not ideal for further metabolic engineering (Fig. 1c and d). Since the synthesis of nucleosides is strictly and complicatedly regulated in the cell, it is difficult to achieve the proper balance between cell growth and nucleoside production using traditional metabolic engineering. Therefore, it is necessary to adopt a rationally designed engineering strategy to search for optimal targets to resolve the problem.

### In silico prediction of novel targets for the bi-level optimization between cell growth and IMP production

**Fig. 2:**
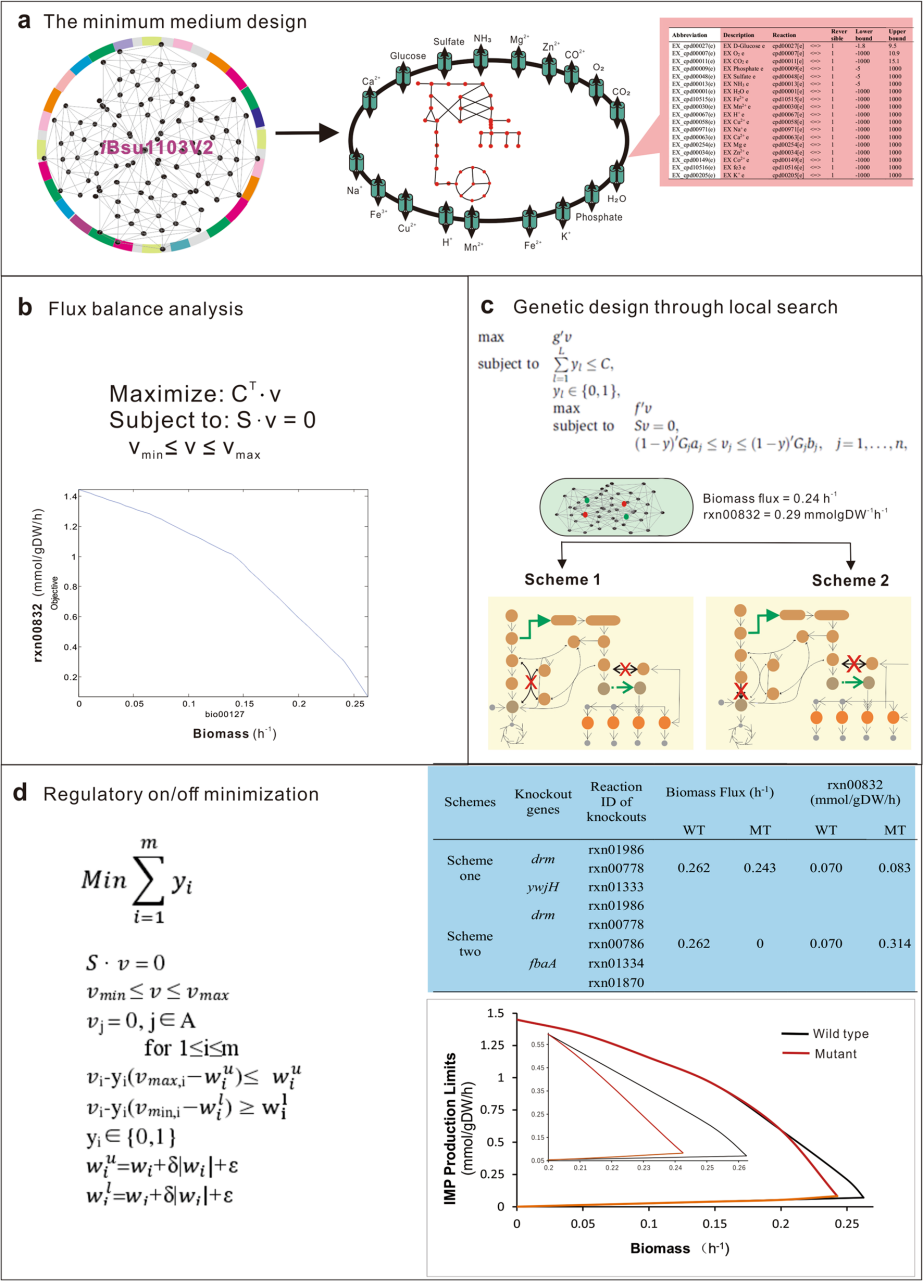
Applications of the genome-scale metabolic network models (GEM) to predict the optimal targets. **a** The minimum medium design through GEM. The minimum medium was designed in the *B. subtilis* model for the simulation calculation. **b** Analysis of IMP flux and Biomass flux by FBA. **c** Prediction of the potential targets by GDLS. **d** Flow disturbance analysis of the mutants by ROOM. Maximal and minimal IMP production rates were predicted at different growth rates

Because IMP is the essential precursor of all purine nucleosides, the relationships between nucleoside flux and biomass as well as IMP productivity were investigated by metabolic flux balance analysis (FBA) based on the *i*Bsu1103V2 model (Additional file 1: Table S3). Through constraint-based FBA simulations, the maximum theoretical growth rate, the synthesis rate of IMP, and the yield of IMP were estimated as 0.263 h^−1^, 1.450 mmol/gDW/h, and 0.806 mol/mol (IMP/glucose), respectively (Fig. 2a). Robustness analysis was further conducted to evaluate the interaction relationship between the metabolic flux of IMP synthesis and cell biomass. The results showed that the synthesis rate of IMP decreased with a continuous increase in the cell growth rate, indicating a competitive relationship between biomass and IMP synthesis. The highest IMP production would result in biomass being zero (Fig. 2b). These results suggested that a rational engineering strategy is required to balance the flux distribution between biomass and IMP production.

Considering the complexity of gene–protein–reaction relationship in the glycolysis, gluconeogenesis, the nucleotides terminal pathways (purine/pyrimidine conversions) and the transport systems (Additional file 2: Tables S2–S4), genetic design through local search (GDLS) was used to predict the optimal targets for the bi-level optimization between cell growth and IMP production (Fig. 2c). The reaction formulas catalyzed by enzymes from the EMP, the PPP and the purine synthesis pathway were selected as potential targets to stimulate IMP production. Because of a competitive relationship between the metabolic fluxes of IMP and biomass, only two strategies were obtained to strengthen the metabolic flux of IMP to the maximal level of 0.293 mmol/gDW/h. Specifically, strategy 1 was designed to knock out the pentose phosphate mutase encoded by the *drm* gene and the transaldolase encoded by the *ywjH* gene, while strategy 2 was designed to knock out *drm* and the fructose bisphosphate aldehyde carboxylase encoded by *fbaA* (Additional file 1: Table S4).

**Fig. 3:**
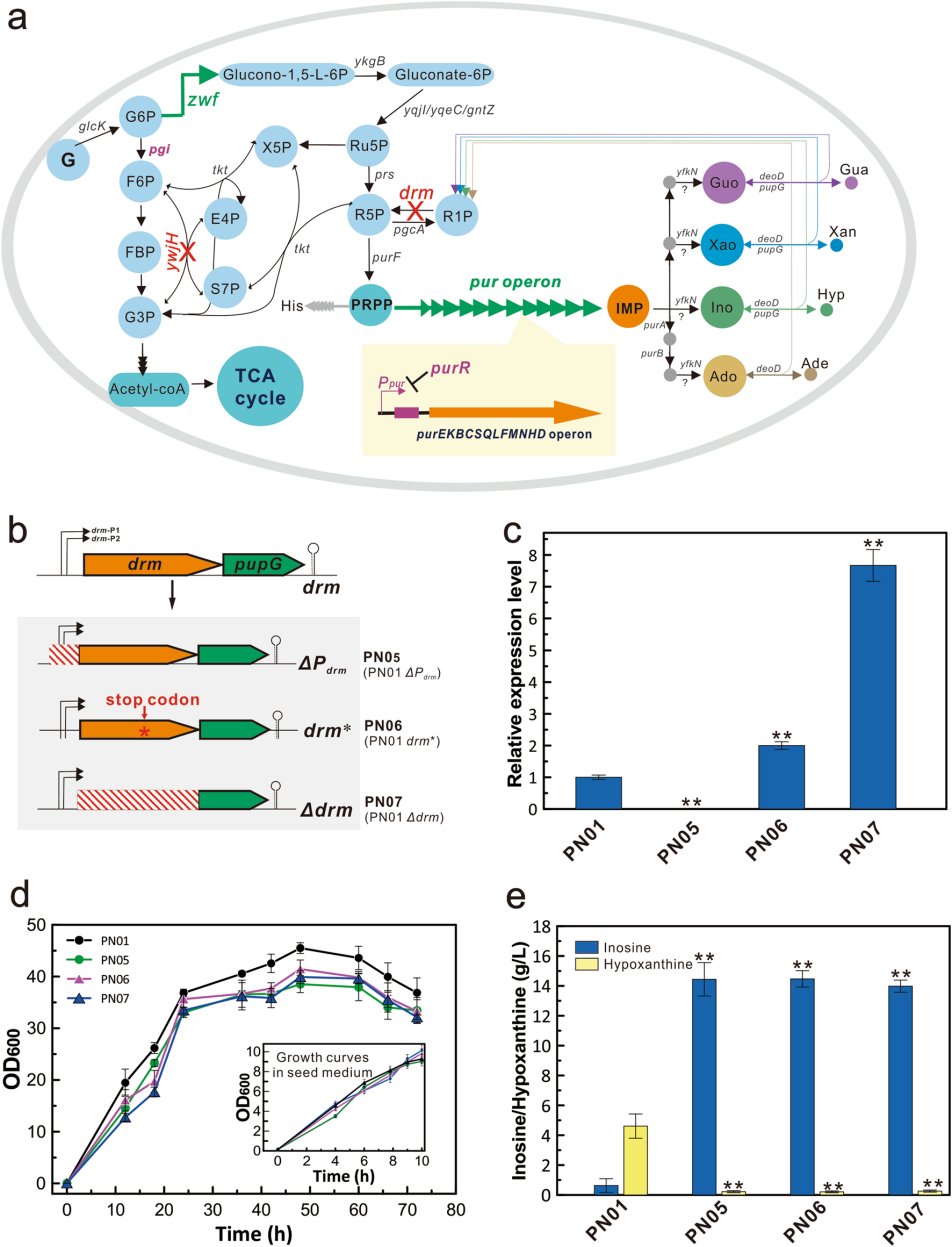
The performance of the Drm-inactivated strains. **a** The engineering strategy proposed by GEM for de novo synthesis of purine nucleosides in *B. subtilis*. Genes/reactions in red indicate deletion and genes/reactions in green indicate overexpression. *glcK*, encoding glucokinase; *zwf*, encoding glucose-6-phosphate 1-dehydrogenase; *ykgB*, encoding 6-phosphogluconolactonase; *pgi*, encoding glucose-6-phosphate isomerase; *ywjH*, encoding transaldolase; *tkt*, encoding transketolase; *prs*, encoding ribose-phosphate pyrophosphokinase; *purF*, encoding amidophosphoribosyltransferase; *drm*, encoding pentose phosphate mutase; *pgcA*, encoding phosphoglucomutase; *purR*, Pur operon repressor. **b** The schematic diagram for the modifying strategies of *drm* inactivation. The engineered strains PN05, PN06, and PN07 were constructed by nonsense point mutation of the *drm* gene, deletion of the promoter *P*_*drm*_, and knockout of the open reading frame, respectively. **c** The relative mRNA expression level of *pupG* gene in the engineering strains. The relative transcriptional levels were analyzed by quantitative real-time PCR using PN01 as the control. **d** The cell growth during shake-flask cultivation. The inset shows the growth curves in the seed medium. **e** The effect of the *drm* deficiency on the production of inosine and hypoxanthine. All error bars indicate ± SD, *n* = 3. A value of *P* less than 0.05 was regarded to be a significant difference with the control strain PN01 using the T-test (**, *P* < 0.01)

Since the predicted targets catalyze other reactions at the same time, whether they could be used as ideal targets for enhancing IMP flux was further analyzed by regulatory on/off minimization (Additional file 1: Table S5). First, the upper and lower bounds of reactions catalyzed by the predicted target were set to "0" using the ChangeRxnBounds command (Fig. 2d). Subsequently, the minimum switch adjustment algorithm (ROOM) was used to assess the effects of the two strategies on biomass formation and IMP production by MATLAB software. Results of these analyses showed that Strategy 2 could remarkably increase the IMP flux by 3.49-fold (from 0.070 to 0.314 mmol/gDW/h), but the growth rate of the mutant strain dropped to zero. Strategy 1 could increase the IMP flux by 18.57% (from 0.070 to 0.083 mmol/gDW/h). More importantly, the growth rate of the mutant strain was only reduced to 0.243, a slight decrease of 7.60% compared to that of the wild-type strain (Fig. 2d). As shown in the metabolic pathway, the predicted targets Drm and YwjH of Strategy 1 are the key backflow nodes of the purine nucleotides to the PPP and PPP to the EMP, respectively (Fig. 3a). In combination with the possible targets for overproduction of IMP and inosine simulated using OptForce_MUST_ (Additional file 2: Table S4), the first backflow node Drm and the purine operon were selected as a new combination to rationally optimize the purine synthesis pathway. Furthermore, the second backflow node YwjH and the glucose 6-phosphate dehydrogenase Zwf were combined to increase the metabolic flow of PPP, which could supply more carbon flux for the synthesis of purine nucleotides.

### Blocking the degradation of purine nucleosides into PPP by inactivating the backflow node Drm

To validate the effects of in silico-predicted targets on the degradation of purine nucleosides, the *drm* gene was genetically modified by promoter knockout, nonsense mutation, and ORF (opening reading frame) deletion to construct the engineered strains PN05 (PN01 *ΔP*_*drm*_), PN06 (PN01 drm*) and PN07 (PN01 *Δdrm*), respectively (Fig. 3b). Since the *drm* and *pupG* genes are located in the same operon, these three mutations might have different effects on *pupG* expression and were subsequently detected by real-time quantitative reverse transcription PCR (qRT-PCR). The mRNA expression levels of the *pupG* gene of the PN06 and PN07 strains were upregulated 2- to 7-fold compared to the original strain PN01, but it could not be detected in PN05 due to promoter deficiency (Fig. 3c). Compared with the nonsense mutation, deleting the *drm* gene considerably increased the expression of the *pupG* gene due to the shortened distance between the promoter and ORF.

The growths of the PN05, PN06, and PN07 strains were marginally lower than that of the original strain PN01 in shake-flask cultures, while similar growth was observed in seed cultures (Fig. 3d). These results indicated that Drm inactivation did not significantly affect cell growth. The inosine titers of the PN05, PN06, and PN07 strains reached 13.98–14.47 g/L with improved yields (0.085–0.090 mol/mol glucose), productivities (0.194–0.201 g/L/h) and synthesis rates (0.064 to 0.064 mmol/gDW/h), notably increasing above 20-fold compared to that of the original strain PN01 (Fig. 3e and Additional File 2: Table S1). Interestingly, Drm inactivation in different ways remarkably reduced hypoxanthine synthesis from 4.61 to 0.21 g/L.

To clarify the effects of Drm and PupG on the degradation of purine nucleosides, complementary experiments were carried out by separately expressing the *drm* and *pupG* genes in PN05 (Additional file 1: Fig. S2). The *pupG* expression strain PN05-s2 (PN05 *lacA::P*_*xyl*_* -pupG -xylR*) produced high inosine and low hypoxanthine, which were similar to the control strains PN05 and PN05-s0 (PN05 *lacA::P*_*xyl*_* -xylR*). In contrast, dramatically decreased inosine titer and increased hypoxanthine production were detected in the *drm* expression strain PN-5-s1 (PN05 *lacA::P*_*xyl*_* -drm-xylR*). Combined with the above results, Drm, not the purine nucleoside phosphorylase PupG, exhibited a major effect on increasing inosine production and decreasing its degradation (Fig. 3e and Additional file 1: Figure S2). Drm is thus a key backflow node for blocking the degradation of purine nucleosides into the PPP and promoting inosine accumulation. Therefore, the in silico-predicted target can effectively improve the biosynthesis of purine nucleosides without obvious impact on cell growth compared to traditional engineering targets (Fig. 1).

### Releasing complex regulation of the purine operon to increase the synthesis of purine intermediates

**Fig. 4:**
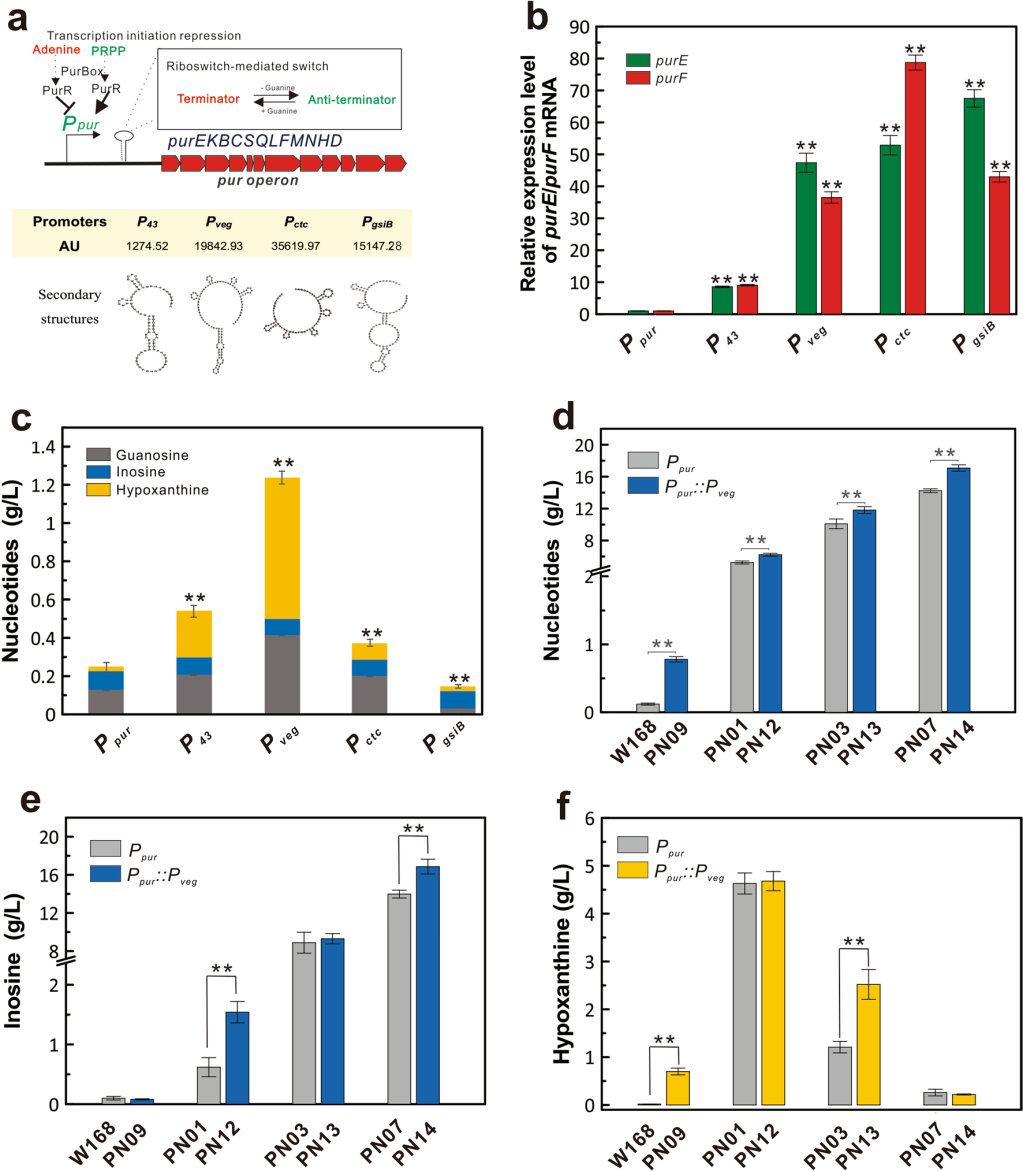
The replacement of promoter *P*_*pur*_ to release feedback regulation of key enzymes in purine synthesis. **a** Different strength of promoters selected to release the transcription initiation repression on the *pur* operon. The translation initial efficiencies of promoters are predicted by RBS calculator v1.1 [27]. The secondary structures formed between 5’-UTR and *purE* are predicted by DNAMAN. **b** The relative mRNA expression level of *purE* and *purF* genes under different promoters. The relative transcriptional levels are analyzed by quantitative real-time PCR using *P*_*pur*_ as the control. **c** The effect of promoter replacement on the accumulation of purine intermediates in the W168 strain. **d** The effect of the promoter replacement on the accumulation of nucleotides in inosine engineered strains. **e** The accumulation of inosine in the engineered strains. **f** The accumulation of hypoxanthine in the engineered strains. The promoter *P*_*veg*_ was used to replace *P*_*pur*_ in the strains W168, PN01, PN03, and PN07, separately producing strains PN09 (W168 *P*_*pur*_::*P*_*veg*_), PN12 (W168 Δ*purA P*_*pur*_::*P*_*veg*_), PN13 (W168 Δ*purA* Δ*pupG P*_*pur*_::*P*_*veg*_), and PN14 (W168 Δ*purA* Δ*drm P*_*pur*_::*P*_*veg*_). All error bars indicate ± SD, *n* = 3. A value of *P* less than 0.05 was regarded to be a significant difference from the original promoter *P*_*pur*_ (*, *P* < 0.05; **, *P* < 0.01)

The purine operon is strictly regulated by transcription initiation repression and ribosome-mediated switch in the cell (Fig. 4a). Optimization of the purine operon by promoter replacement was used to increase purine nucleoside synthesis. After analysis by DNAMAN and RBS calculator v1.1 [27], promoters of different strengths (*P*_*43*_, *P*_*veg*_, *P*_*ctc*_ and *P*_*gsiB*_) with different secondary structures were selected to replace the original promoter P_*pur*_ of the purine operon. Among these promoters, *P*_*ctc*_ formed the least stem–loop structure with the highest translation initiation efficiency of 35619.97 AU. The stem–loop structure of *P*_*veg*_ is far from the ribosome binding site (RBS) and the start codon with an initiation efficiency of 19842.93 AU. The promoters *P*_*43*_ and *P*_*gsiB*_, close to RBS and the initiation codon, formed a relatively complex stem–loop structure, which could result in a negative impact on translation initiation. *P*_*43*_ forming the most stable stem–loop structure, had the lowest translation initiation efficiency of 1274.25 AU, whereas *P*_*gsiB*_ had a translation initiation efficiency of 15147.28 AU.

To detect the effects of different promoters on the transcription levels of the purine operon and the synthesis of purine intermediates, the promoter *P*_*pur*_ in wild-type strain W168 was separately replaced by *P*_*43*_, *P*_*veg*_, *P*_*ctc*_ and *P*_*gsiB*_. The mRNA levels of the *purE* and *purF* genes were all upregulated 8- to 78-fold under the control of the replaced promoters, suggesting that the transcription levels of the purine operon were enhanced by promoter replacement (Fig. 4b). Purine intermediates of inosine, guanosine, and hypoxanthine were increased in the mutant strains (Fig. 4c). The promoter *P*_*veg*_ produced the highest accumulation of 1.24 ± 0.10 g/L purine intermediates, a 4.93-fold improvement in comparison with that of the W168 strain (0.25 ± 0.07 g/L). Therefore, *P*_*veg*_ could efficiently relieve the complex regulation of the purine operon and dramatically enhance the synthesis of purine nucleotides, which was selected to optimize the inosine engineered strains.

To demonstrate the effects of promoter replacement on inosine production, *P*_*pur*_ in the engineered strains was replaced by *P*_*veg*_ to generate PN09 (W168 *P*_*pur*_::*P*_*veg*_), PN12 (PN01 *P*_*pur*_::*P*_*veg*_), PN13 (PN03 *P*_*pur*_::*P*_*veg*_) and PN14 (PN07 *P*_*pur*_::*P*_*veg*_). Flask cultivation showed that purine intermediates of inosine and hypoxanthine were all significantly increased in the inosine engineered strains (*P* < 0.01), further demonstrating that *P*_*veg*_ could efficiently enhance the synthesis of purine nucleotides (Fig. 4d). However, inosine production of PN09 and PN13 did not significantly increase compared to their original strains W168 and PN03 (W168 Δ*purA* Δ*pupG*), respectively (Fig. 4e). The accumulation of hypoxanthine increased by 1–35 fold in these strains, suggesting that the enhanced inosine was degraded to hypoxanthine (Fig. 4f). When *P*_*pur*_ in PN01 (W168 Δ*purA*) and PN07 (W168 Δ*purA* Δ*drm*) strains were replaced with *P*_*veg*_, the inosine production of PN12 and PN14 remarkably increased by 148% and 21%, respectively (Fig. 4e). The accumulations of hypoxanthine in PN12 and PN14 were similar to their original strains (Fig. 4f). Further improvement in the in silico-designed strain PN07 significantly increased inosine titer to 16.86 ± 0.78 g/L with improved yield (0.103 mol/mol glucose), productivity (0.234 g/L/h) and synthesis rate (0.074 mmol/gDW/h), but the same modification was not effective in the traditionally engineered strain PN03 (Fig. 4e and Additional file 2: Table S1).

### Enhancing the PPP flow by blocking the backflow node YwjH and overexpressing the key enzyme Zwf

**Fig. 5:**
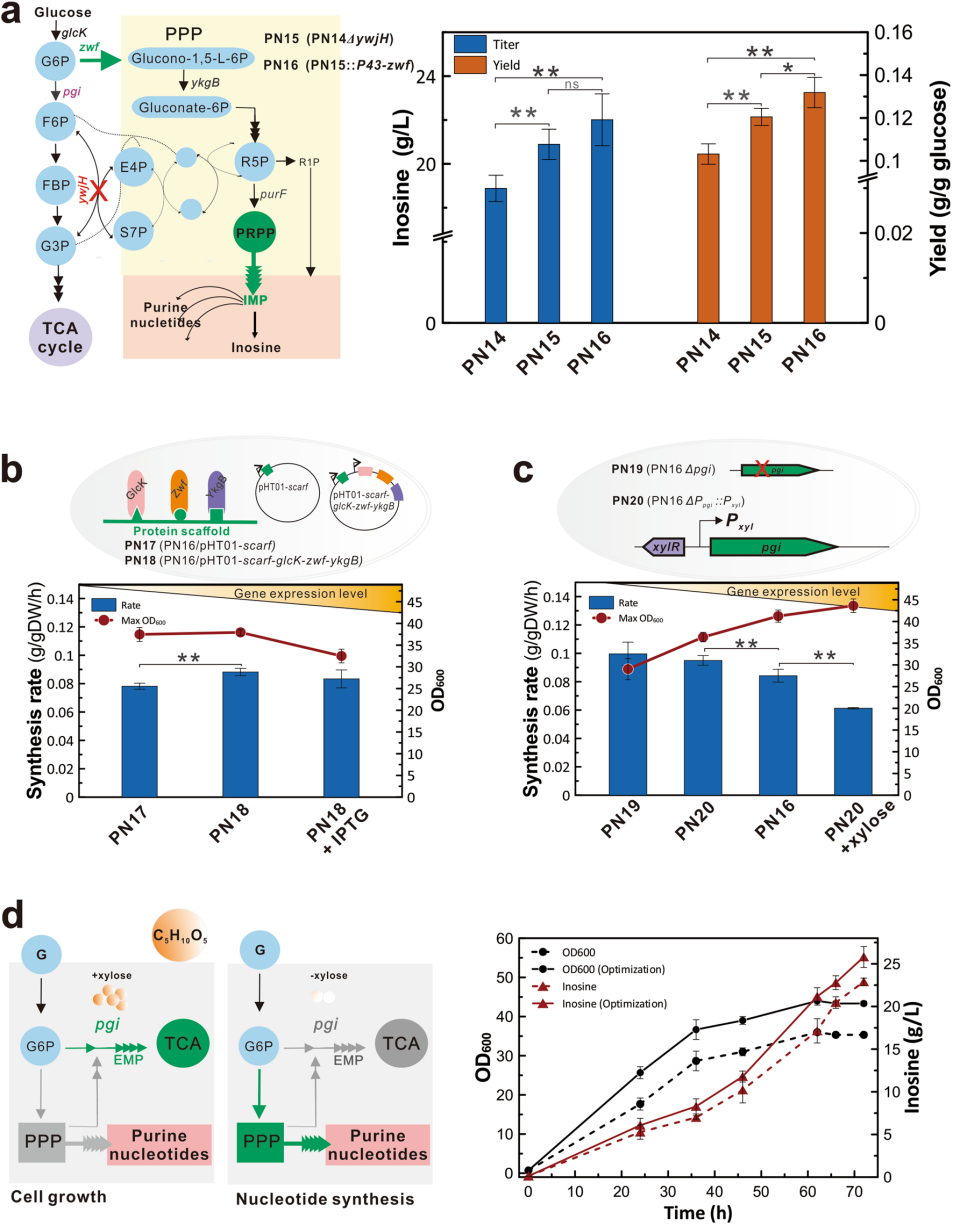
Overproduction of inosine by enhancing the metabolic flow of PPP. **a** Inosine productions of the engineered strains by knocking out the *ywjH* gene and overexpressing the *zwf* gene. **b** The effect of linearly assembling proteins GlcK, Zwf, and YkgB on the inosine synthesis and cell growth. The protein scaffolds GBD, SH3, and PDZ were separately used to linearly assemble proteins GlcK, Zwf, and YkgB. **c** The effect of the *pgi* expression level on the inosine synthesis and cell growth. Conditional expression of the *pgi* gene under the control of the *P*_*xyl*_ promoter was used to balance the inosine synthesis and cell growth. **d** Two-stage fermentation in the inosine engineered strain by the *pgi*-based metabolic switch. All error bars indicate ± SD, *n* = 3. A value of *P* less than 0.05 was regarded to be a significant difference with the W168 strain using the T-test (*, *P* < 0.05; **, *P* < 0.01)

Based on the guidance of GEM, there is a complex exchange of metabolic flux between the EMP and the PPP. To supply more carbon flux for the purine synthesis, the flow of PPP to EMP could be weakened by inactivating the backflow node YwjH. Meanwhile, the flow of glucose to the PPP could be strengthened by regulating the expression of key enzymes (Fig. 5a). Based on this, the glucose-6-phosphate would be effectively catalyzed by the glucose 6-phosphate dehydrogenase encoded by the *zwf* gene to enter into the PPP, and therefore supply more carbon flux for the synthesis of purine nucleotides. According to the in silico design, the *ywjH* gene was knocked out to generate the engineered strain PN15 (PN14 Δ*ywjH*). The inosine titer of PN15 was significantly increased to 20.89 ± 0.69 g/L without an apparent reduction in cell growth (Fig. 5A and Additional file 1: Figure S3). This result indicated that YwjH inactivation could significantly promote nucleoside biosynthesis, in accordance with the in silico prediction. To further strengthen the metabolic flow of the PPP, the *zwf* gene controlled by a strong promoter was integrated into the genome to generate strain PN16 (PN15:: *P*_*43*_-*zwf*), which increased inosine titer to 22.01 ± 1.18 g/L with improved yield (0.132 mol/mol glucose, *p* = 0.024), productivity (0.306 g/L/h) and synthesis rate (0.084 mmol/gDW/h, *p* = 0.038) (Fig. 5a and Additional File 2: Table S1). Therefore, the synthesis of purine nucleosides was considerably improved by blocking the essential backflow node and overexpressing the key enzyme Zwf to enhance the PPP flux.

### Efficient production of inosine by dynamically switching the metabolic flux of biomass and product

To coordinate biomass and desired metabolites, a dynamic switch was constructed to allocate the metabolic flux into the EMP and PPP. Two regulation strategies were designed to dynamically control the flow of glucose 6-phosphate into the EMP or PPP. In the flow node of glucose to the PPP, the scaffold proteins GBD, SH3 and PDZ were separately used to linearly assemble three key enzymes encoded by the *glcK*, *zwf* and *ykgB* genes (Fig. 5b). In the absence of IPTG, the engineered strain PN18 (PN16/pHT01-scaf-*glcK*-*zwf*-*ykgB*) showed an improved synthesis rate without an obvious impact on cell growth in comparison with those of the control strain PN17 (PN16/pHT01-scaf). In the presence of an inducer, the strain PN18 showed a similar inosine synthesis rate and a slight reduction in cell growth (Fig. 5b and Additional file 1: Figure S4). Taken together, cell growth and inosine synthesis did not relate to the gene expression level, suggesting that the linear enzyme system was not an ideal candidate as a switch.

To dynamically control the metabolic flow of glucose into the EMP, the *pgi* gene was knocked out or conditionally expressed by the inducible promoter *P*_*xyl*_ (Fig. 5c). Flask cultivation of the engineered strains PN19 (PN16*Δpgi*) and PN20 (PN16 *P*_*pgi*_::*P*_*xyl*_) showed that *pgi* deficiency greatly inhibited cell growth (Fig. 5c and Additional file 1: Figure S5). Compared to the original strain PN16, the strain PN20 showed significantly decreased cell growth and enhanced inosine biosynthesis in the absence of xylose (*P* < 0.01). In contrast, cell growth was significantly enhanced and the inosine biosynthesis significantly decreased in the presence of xylose (*P* < 0.01, Fig. 5c). These results showed that *pgi* expression controlled by an inducer was effective for regulating cell growth and inosine synthesis. The low expression level of *pgi* likely weakened central carbon metabolism, which could further divert metabolic flux to inosine synthesis (Fig. 5c). While, overexpression of *pgi* by the addition of an inducer could enhance central carbon metabolism, which would promote cell growth and thereby decrease inosine synthesis (Fig. 5C). Therefore, *P*_*xyl*_-driven *pgi* was used to optimize the fermentation process by dynamically regulating biomass and inosine synthesis.

According to the above results, a two-stage fermentation based on the metabolic switch was further adapted to improve inosine production. While the *pgi* gene was conditionally controlled by *P*_*xyl*_, the cell growth was improved as the inducer concentration increased from 0 to 3% (Additional file 1: Figure S6). In the cell growth stage, xylose was added to increase *pgi* expression, and thereby enhance the EMP flow to promote biomass. In the inosine production stage, *pgi* expression was reduced to weaken the EMP flow by removing the inducer, resulting in the flow enhancement of the PPP and purine nucleosides (Fig. 5d). Under the optimized fermentation process, the final engineered PN20 produced up to 25.81 ± 1.23 g/L inosine with a yield of 0.126 mol/mol glucose, a productivity of 0.358 g/L/h and a synthesis rate of 0.088 mmol/gDW/h in shake-flask cultivation (Additional file 2: Table S1). Similar production performances were also observed in a 5-L fed-batch cultivation (Additional file 1: Figure S8). Therefore, the metabolic switch was successfully developed to dynamically regulate metabolic flow and maximize inosine production.

### Construction of the universal purine chassis strain

**Fig. 6:**
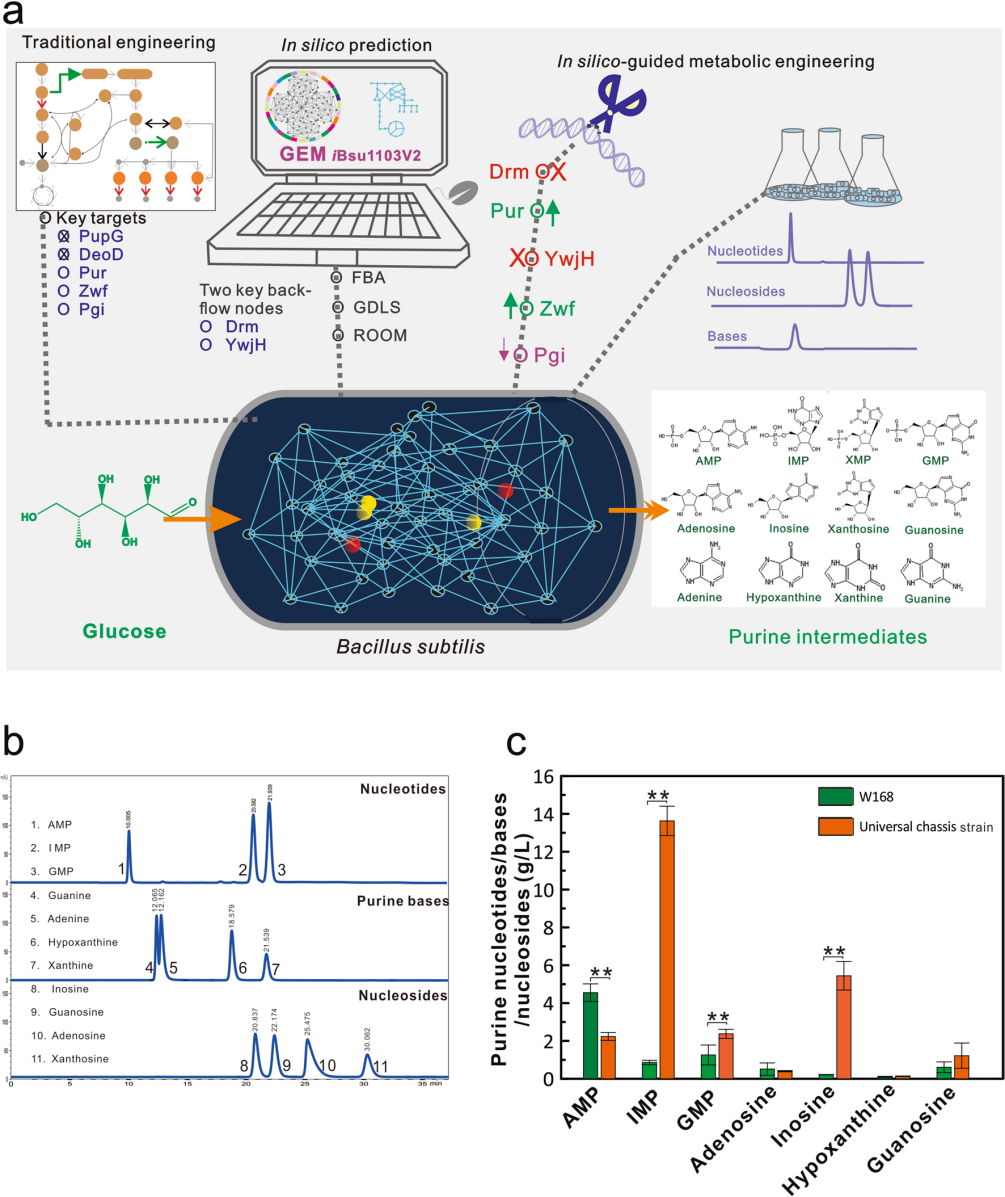
Construction of the general purine chassis bacterium by in silico-guided metabolic engineering strategy. **a** Schematic diagram for in silico-guided metabolic engineering strategy. **b** The retention times of purine nucleosides, bases, and nucleosides by HPLC. **c** Determination of purine nucleosides metabolites synthesized in the general purine chassis strain. All error bars indicate ± SD, *n* = 3. A value of *P* less than 0.05 was regarded to be a significant difference with the W168 strain using the T-test (*, *P* < 0.05; **, *P* < 0.01)

Under the guidance of in silico design by GEM, the synthesis of inosine was remarkably improved by blocking the backflow nodes, releasing feedback inhibition of the purine operon and regulating the expression of key enzymes (Fig. 6a). Here, a general chassis bacterium for efficient synthesis of various purine intermediates was constructed by expressing the *purA* gene in the finally optimized strain. The purine metabolites of the universal chassis strain were detected by HPLC after flask cultivation. The detection condition of each standard was optimized to separate each purine base, nucleotide, or nucleoside well (Fig. 6b). Purine intermediates of AMP, IMP, GMP, adenine, hypoxanthine, guanine, inosine, and guanosine were detected in the universal chassis strain (Fig. 6c). Among these metabolites, the accumulations of IMP, GMP, inosine and guanosine were remarkably increased in PN17, whereas AMP and adenosine were reduced. As a decomposition product of inosine, the hypoxanthine concentration was extremely low. The accumulation level of purine intermediates was ranked as IMP > inosine > AMP > GMP > guanosine > adenosine > hypoxanthine. Among these metabolites, the high IMP titer of 13.63 ± 0.78 g/L provided a sufficient precursor for the synthesis of various purine intermediates. Therefore, the universal chassis strain was successfully constructed to produce various purine intermediates under the guidance of an in silico-guided engineering strategy, providing an effective and universal approach for metabolic engineering of the purine biosynthesis pathway.

## Discussion

In this study, a rationally designed strategy based on the genome-scale metabolic network model succeeded in maximizing the biosynthesis of purine intermediates and biomass. With advantages over previous studies [6, 7, 9, 11, 13, 14], the method developed here investigates the global regulation and metabolic flux to construct a general purine chassis strain for the first time and achieves the highest yield of inosine in de novo engineered bacteria to our knowledge. Based on the metabolic pathway, purine nucleosides are not the end products, but can be converted into ribose and base by the salvage pathway, resulting in a decline of nucleoside accumulation [4]. In bacterial cells, the decomposition of purine nucleosides is mainly reversibly catalyzed by the purine-nucleoside phosphorylase PNP (encoded by *deoD* or *pupG* genes) (Fig. 3a). In previous studies, *deoD* and/or *pupG* were normally knocked out to increase the accumulation of purine nucleosides [13, 14]. However, the biomass was usually reduced dramatically in the engineered strains, which was difficult to further optimize [11]. Consequently, the lack of understanding global regulation and metabolic networks results in poor cell performance and low production [17]. Therefore, it is necessary to block the conversion of nucleosides into other substances without affecting cell growth as much as possible. Based on the computational prediction, the Drm enzyme was found to be an essential backflow node of the purine synthesis pathway toward PPP. The present study first proved that Drm deficiency could preferentially block the decomposition of nucleosides and considerably increase the production of purine nucleosides without an obvious impact on cell growth (Fig. 2). To validate the predicted target, the *drm* gene was inactivated in different ways since the *drm* and *pupG* genes are located in the same operon in *B. subtilis*. Although the mRNA expression levels of the *pupG* gene were different in these *drm* inactivation strains, the inosine titers increased to 13.98–14.47 g/L. These results indicate that the *drm* gene plays a major role in blocking inosine decomposition, which is further verified by the complementary experiment (Additional file 1: Figure S1). Therefore, we show for the first time that the loss of Drm function can effectively block the decomposition of purine nucleosides.

The purine synthesis pathway is strictly regulated by complex regulatory mechanisms, coupled with amino acids, folic acids and energy metabolism [28]. To effectively increase nucleoside synthesis, it is particularly important to release the complex regulations of the purine operon (Fig. 3a). The *pur* operon (*purEKBCQLFMNHD*) encoding 11 enzymes is necessary for de novo synthesis of the precursor IMP of the purine intermediates in *B. subtilis*. However, transcription of this operon is dually regulated by a repressor protein encoded by the *purR* gene [29] and a riboswitch controlled by purine levels [16]. The regulatory sequences are located in the intergenic sequence upstream of the operon. Usually, the expression level of the purine operon is increased by deleting the *purR* gene, destroying the riboswitch structure, or optimizing the core sequence of the *pur* operon promoter region [3, 13, 30]. In this study, the promoter sequence of the purine operon was replaced with constitutive promoters of different strengths (including P_*43*_, P_*veg*_, P_*ctc*_, and P_*gsiB*_) to eliminate the *pur* box sequence and riboswitch structure of the PurR recognition site in the original promoter region, which can simply and effectively relieve complex regulation. Combining the transcription level and translation initiation efficiency (Fig. 4a, b), the highest expression level of the purine operon was in the W168 strain with P_*ctc*_, followed by P_*veg*_ or P_*gsiB*_, and the lowest was P_*43*_. However, P_*veg*_ reached the highest nucleoside accumulation in the W168 (Fig. 4c), possibly attributing to the existence of the post-transcriptional/translational modification in the regulation of the purine operon [31]. Consequently, inosine titer was significantly increased to 16.86 ± 0.78 g/L in the inosine engineered strain by replacing P_*pur*_ with P_*veg*_ (Fig. 4d).

Due to the complexity of the purine metabolic network, the metabolic flow of nucleosides might be difficult to enhance through traditional metabolic engineering [15]. First, the PPP, the carbon carrier pathway for purine nucleotide synthesis, is not the mainstream pathway in cells and the metabolic flow is relatively low [11]. Second, when the metabolites of PPP increase, they will return to the central carbon metabolism pathway (Fig. 5a). Based on these reasons, GEM was used to predict the essential target transaldolase YwjH, which can reversibly catalyze erythrose-4-phosphate (E4P) and sedoheptulose-7-phosphate (S7P) in PPP to form fructose-6-phosphate (F6P) and pyruvate in EMP. According to the analysis of metabolic network, YwjH deficiency blocks the PPP flow to the EMP. Additionally, overexpression of transaldolase has been reported to affect the synthesis of ribose-5-phosphate (R5P) and increase histidine accumulation [32]. In contrast, transaldolase deficiency could weaken the competitive pathways and promote the synthesis of ribose-5-P. The metabolic flow of PRPP synthesis was thereby enhanced, contributing to the accumulation of purine nucleosides. As a result, *ywjH* knockout significantly increased inosine titer to 22.01 ± 1.18 g/L (Fig. 5a).

The performance of the inosine engineered strain was further optimized to balance cell growth and inosine production by constructing a dynamic switch. Glucose is phosphorylated by the glucokinase GlcK to synthesize glucose-6- phosphate (G6P), which can be catalyzed either by glucose-6-P-1-dehydrogenase Zwf to form glucono-1,5-L-6P of PPP or by glucose-6-P isomerase Pgi to form F6P of EMP (Fig. 3a). Previously, the protein scaffolding strategy was designed by the principle of interaction between scaffold proteins and their ligands to combine multiple targets on the metabolic network into a linear pathway and rebuild a multi-enzyme-catalyzed biological reaction system in the cell [33]. This technology has been successfully applied to the accumulation of target products such as mevalonic acid, saccharic acid, alkanes, and butanediol [34–37]. To dynamically control the metabolic flow of PPP and EMP, in the present study, the scaffold proteins GBD, SH3, and PDZ were separately used to linearly assemble *glcK*, *zwf*, and *ykgB* expressed by the inducible promoter P_*grac*_ (Fig. 5b). Inosine production did not change under the control of the inducer except for the reduced biomass by the addition of the inducer, suggesting an inefficient effect on the dynamic control of metabolic flow. Then, the *pgi* expression level controlled by the inducible promoter P_*xyl*_ was used to establish an efficient dynamic switch (Fig. 5c). Notably, inosine production was greatly decreased by overexpressing the *pgi* gene under the induction condition, but was increased in the absence of an inducer. Meanwhile, the cell growth was improved by adding an inducer, but was decreased by removing the inducer, suggesting a potential switch to dynamically regulate the biomass and product. The expression intensity of the *pgi* gene was further adapted as an effective metabolic switch to balance the product and biomass, achieving inosine titers of 25.81 ± 1.23 g/L and 26.6 g/L by shake-flask and fed-bath cultivation, respectively (Fig. 5d and Additional file 1: Figure S7). HPLC data showed that a large amount of inosine and small amounts of other metabolites were detected in the fermentation products, suggesting low concentrations of overflow metabolites. At this stage, we cannot rule out that other factors, such as the expression intensity of the multi-enzyme catalytic system and the ratio of each enzyme’s expression level might also affect inosine synthesis. It is necessary to finely control the expression levels of the key enzymes in the metabolic pathway to achieve the joint and cooperative expression of multiple genes in further studies [38].

Based on the robustness analysis by constraint-based FBA, a competitive relationship between biomass and IMP synthesis suggests a rational engineering strategy is urgently required. Furthermore, many enzymes are responsible for multiple reactions in the glycolysis, gluconeogenesis, the nucleotides terminal pathways (purine/pyrimidine conversions) and the transport systems (Additional file 2: Table S4). Effective targets in these pathways are difficult to predict by OptForce_MUST_, which identifies genetic modifications through maximizing the objective flux [39]. Therefore, GDLS and ROOM were used to analyze, predict, and assess the optimal targets through the bi-level optimization between cell growth and IMP production (Fig. 2c) [25, 26]. The predictions by FBA, GDLS, and ROOM were all consistent with experimental results in this study (Additional file 1: Table S6). First, the shake-flask and fed-batch cultivation experiments using FM showed that the final engineered strain achieved 0.172–0.237 h^−1^ of cell growth rate and 0.081–0.096 mmol/gDW/h of inosine synthesis rate, closing to predictions of ROOM (0.243 h^−1^of biomass and 0.083 mmol/gDW/h of IMP flux). Furthermore, the fermentation experiment was carried out using the minimum medium (MM) that was usually used for in silico design (Additional file 1: Figure S8, Additional file 2: Table S1). The titer, yield, and productivity of MM were lower than that of fermentation medium (FM), but its inosine synthesis rate (0.220 mmol/gDW/h) was remarkably higher than that of FM (0.081–0.096 mmol/gDW/h). The inosine synthesis rate of MM was close to the maximal value of 0.293 mmol/gDW/h predicted by GDLS (Additional file 1: Table S6). Moreover, the specific growth rate of MM (0.011 h^−1^) was dramatically lower than that of FM (0.172–0.237 h^−1^). These experimental data showed that the higher IMP flux would result in lower biomass in MM, indicating a competitive relationship between biomass and IMP flux as predicted by FBA. Compared to the glucose minimal medium, the rich medium has considerably improved the biomass of the final engineered strain, which still has the potential to maximize the product synthesis rate by fed-batch fermentation in further study.

## Conclusions

In the present study, GEM was used to in silico predict metabolic targets for optimization of purine nucleoside synthesis. The production of purine nucleosides was remarkably improved by optimizing the targets *drm*, P_*pur*_, *ywjH*, *zwf*, and *pgi* to block nucleoside degradation, strengthen the PPP, and weaken the EMP. Under a two-stage fermentation by a *pgi-*based metabolic switch, the engineered strain produced up to 25.81 ± 1.23 g/L inosine without affecting cell growth, suggesting the highest inosine production in de novo engineered bacteria to date. Finally, an in silico-guided metabolic engineering strategy was successfully used to generate a universal chassis bacterium capable of producing various purine intermediates, such as inosine, adenosine, guanosine, IMP, and GMP. Overall, the rational engineering strategy based on GEM is successfully used to resolve the competitive relationship between cell growth and nucleoside synthesis, suggesting potential applications for a wide range of biotechnology products.

## Methods

### Strains, medium, and chemicals

The strains used in this study are listed in Additional file 1: Table S1. EC135 lacking all R-M systems and orphan MTases was used as a cloning host to construct plasmids [40]. All strains were cultured in Luria–Bertani (LB) medium (10 g/L tryptone, 5 g/L yeast extract, and 10 g/L NaCl) supplemented with the appropriate antibiotics (100 µg/mL ampicillin was used in *E. coli* and 20 µg/mL erythromycin, 10 µg/mL kanamycin, and 10 µg/mL chloromycetin were used in *B. subtilis*).

Kits for DNA purification/gel recovery and extracting genomic DNA, plasmid DNA, and RNA were purchased from TIANGEN Biotech (Beijing, China). DNA polymerase, restriction enzymes, and dNTPs were purchased from New England Biolabs (USA). Antibiotics, inducers, and standard chemicals, such as ampicillin, kanamycin, chloromycetin, erythromycin, IPTG, xylose, purine bases, nucleotides, and nucleosides were purchased from Sigma-Aldrich (USA). Tryptone and yeast extract were purchased from Oxoid Company (UK). Other reagents for cell culture and fermentation medium were all analytical pure, and purchased from Beijing Modern Oriental Fine Chemical Co., Ltd (China).

### DNA manipulation for plasmid and strain construction

The TCCRAS method was used for scarless manipulation of the bacterial genomes as described by our previous studies [41]. To construct recombinant plasmids for gene modifications in *B. subtilis*, the upstream and downstream DNA fragments flanking the targets *purA*, *pupG*, *deoD*, *drm*, *P*_*pur*_, *ywjH*, *pgi*, and *zwf* were amplified using W168 genomic DNA as the template. The purified DNA fragments were jointed by splice overlap extension (SOE) PCR and then ligated with the vector pWYE486 using the digestion–ligation approach or NEBuilder HiFi DNA fragment rapid assembly kit (New England Biolabs, USA). After verification by PCR and DNA sequencing, the recombinant plasmids were separately transformed into competent *B. subtilis* cells to generate engineered strains through two rounds of homologous recombination (Additional file 1: Table S1). The detailed procedures for constructing recombinant plasmids, preparing competent cells, and screening engineered strains were carried out according to previous methods [41, 42]. All of the primers used in this study are shown in Additional file 1: Table S2 and were synthesized by Invitrogen (Shanghai, China). PCR amplification was performed with Q5 DNA polymerase (New England Biolabs, USA) following the procedure of the manufacturer. DNA sequencing was performed by Beijing Genomics Institute (BGI, Beijing, China) and Tianyi Huiyuan Biotechnology Co., Ltd.

### Constraint-based metabolic flux analysis

In silico analysis of metabolic fluxes for the growth rate and IMP/inosine overproduction was performed by the *i*Bsu1103V2 model using commercially available MATLAB software and GLPK solvers [24]. According to our previous method [21], OptForce_MUST_ was used to predict the possible reactions/genes that were required to be knocked out or up/downregulated to maximize the production of IMP/inosine using ^13^C flux data of the wild-type strain on glucose under aerobic condition [43]. The main features of the model are shown in Additional file 1: Table S3. During the simulation calculation, the medium used in the *B. subtilis* model was the minimum medium. The upper and lower limits of the flux for each exchange reaction in the model are set as the previous method [44]. Based on the experimental value [43, 45], the upper limits of uptake rates for glucose, O_2_ and CO_2_ are set to 9.5, 10.9, and 15.1 mmol/gDW/h (millimoles per gram of dry weight per hour), respectively. The lower limits of the absorption rates for phosphate, sulfate, and NH_3_ are set to − 5 mmol/gDW/h. H_2_O, Ca^2+^, H^+^, K^+^, Mg^2+^, Na^+^ and Fe^3+^ are allowed to be freely transported as external metabolites for exchange (Fig. 2a).

Flux balance analysis (FBA) was used to analyze the metabolic flux distribution of *B. subtilis*. The organism was assumed to be in a pseudo-steady state in which the concentration of each intermediate metabolite was unchanged [46]. The calculation principle of FBA can be expressed by the following formula:

1 $$\begin{gathered} {\text{Maximize}}:{\text{ C}}^{{\text{T}}} \cdot {\text{v}} \hfill \\ {\text{Subject to}}:{\text{ S}} \cdot {\text{v }} = \, 0 \hfill \\ {\text{v}}_{{{\text{min}}}} \le {\text{ v }} \le {\text{ v}}_{{{\text{max}}}} , \hfill \\ \end{gathered}$$

where C_T_ is the objective function value, expressed as a function of the flux of each reaction; S is the m × n order stoichiometric matrix; v is the reaction flux; v_max_ is the upper limit flux of each reaction; and v_min_ is the lower limit flux of each reaction. In general, the accumulation rate of biomass was used as an objective function to calculate the metabolic flux distribution in the case of maximizing biomass.

Genetic design through local search (GDLS) converts the bi-level optimization problem (metabolic fluxes of biomass and IMP synthesis) into a mixed-integer linear programming problem using MATLAB software and GLPK (GNU Linear Programming Kit) solvers. An effective, low-complexity multi-path search was carried out to find a set of locally optimal strategies in the solution space that maximizes the target metabolic flow under a certain growth rate of the strain (≧0.05 h^−1^ in this study). Based on the *i*Bsu1103V2 model, most purine nucleosides/nucleotides are predicted to be secreted out of the cell, including AMP, 3'-AMP, xanthosine, guanosine, GMP, guanine, adenosine, adenine and inosine. The initial calculation principle was designed as previously described [25]. The calculation principle of GDLS can be expressed by the following formula:

max $$g^{\prime}v$$.

subject to $$\sum\limits_{l = 1}^{L} {y_{l} } \le C$$,

$$y_{l} \in \left\{ {0,1} \right\}$$,

max $$f^{\prime}v$$,

subject to $$Sv = 0$$,

2 $$(1 - y)^{\prime}G_{j} a_{j} \le \le v_{j} \le (1 - y)^{\prime}G_{j} b_{j} ,\;j = 1, \ldots .n,$$

where g'v is the value of the outer objective function, f'v is the inner objective function, S is the m × n order stoichiometric matrix, v is the reaction flux vector, and G is the GPR relationship graph in the genome metabolic network model to get an l × n matrix composed of genes and reaction equations. G_j_ is the jth column of the matrix G. When the first gene is knocked out, the y_l_ value is one. Otherwise, it is zero. C is the maximum amount of gene knockout allowed.

Regulatory on/off minimization (ROOM) was determined by the smallest number of reactions, which could change the metabolic flow of the modified strain [47]. These reactions would be used as objective functions for the analysis of flow disturbance after metabolic engineering. The calculation principle is as follows:

3 $$\begin{gathered} Min\sum\limits_{i = 1}^{m} {yi} \hfill \\ S \cdot v = 0 \hfill \\ v\min \le v \le v\max \hfill \\ vj = 0,j \in A \hfill \\ {\text{for}}\;{1} \le i \le m \hfill \\ vi - yi(v\max ,i - w_{i}^{u} ) \le w_{i}^{u} \hfill \\ vi - yi(v\min ,i - w_{i}^{l} ) \ge w_{i}^{l} \hfill \\ y_{i} \in \left\{ {0,1} \right\} \hfill \\ w_{i}^{u} = wi + \delta \left| {wi} \right| + \varepsilon \hfill \\ w_{i}^{l} = wi + \delta \left| {wi} \right| + \varepsilon \hfill \\ \end{gathered}$$

where ${\text{Min}}\sum\nolimits_{t = 1}^{m} {y_{i} }$ is the value of the objective function, which is expressed as the total number of reactions that change the metabolic flux. S is the stoichiometric m × n matrix, v represents the reaction flux vector, v_max_ represents the upper limit of each reaction flux, v_min_ represents the lower limit of each reaction flux, and w represents the reaction flux vector of the wild-type strain. The upper threshold is specified by w_i_^u^ and the lower threshold is specified by w_i_^l^.

### Shake-flask cultivation

To determine the production of purine nucleotide/base/nucleoside metabolites, the strains were pre-cultured in seed medium (20 g/L glucose, 10 g/L sodium glutamate, 20 g/L peptone, 20 g/L yeast powder, 5 g/L corn steep liquor, 2.5 g/L NaCl, 1 g/L urea, pH 7.2) at 32 °C and 220 rpm. Until the OD_600_ reached 10–12, three milliliters of seed culture was inoculated into a 500-mL shake flask containing 30 mL of fermentation medium (FM) containing 120 g/L glucose, 16 g/L soybean meal hydrolysate, 14 g/L yeast powder, 15 g/L (NH_4_)_2_SO_4_, 4 g/L MgSO_4_·7H_2_O, 4 g/L K_2_HPO_4_, 0.01 g/L FeSO_4_, 0.1 g/L biotin and 20 g/L CaCO_3_. During the two-stage fermentation process, bacterial strains were first cultured for 12 h in a medium containing 3% xylose, soybean meal hydrolysate, yeast powder, (NH_4_)_2_SO_4_, MgSO_4_·7H_2_O, K_2_HPO_4_, FeSO_4_, biotin and CaCO_3_. Then, the sterilized glucose solution (800 g/L) was supplemented into the culture to a final concentration of 120 g/L. According to our previous method [11], the cells were grown at 36 °C and 220 rpm, and the medium pH was adjusted to 7.0 by supplementation with ammonia. At least three independent experiments were repeated to show the average data, standard deviations and statistical significance.

### Quantitative real-time PCR

Bacterial total RNA was extracted by an RNA isolation kit and NanoDrop 2000c (Thermo Fisher, USA) was used to determine the concentration of RNA sample, which was then subjected to cDNA synthesis using a FastQuant RT Kit. The primers for qRT-PCR are listed in Additional file 1: Table S2. The qRT-PCR experiment was performed using a LightCycler® 96 Real-Time PCR System (Roche, Switzerland). The 16S rRNA gene was used as the reference gene to normalize the mRNA levels of target genes. The negative controls were designed in each PCR to exclude DNA and other contaminants. A melting curve analysis and Rotor-Gene Q series software (Qiagen, Germany) with the 2^−∆∆CT^ method were used to verify and analyze qPCR data [48]. The transcription levels of target genes were determined by the qRT-PCR method. At least three repeated experiments for each sample were carried out.

### Analytical methods

Cell density was measured by determining the absorbance at 600 nm (OD_600_) using a spectrophotometer (V-1100D; Mapada Instruments, Shanghai, China). The concentration of glucose was assayed using an enzyme electrode analyzer (SBA-40D; Institute of Biology, Shandong, China). After dilution and filtration of the culture supernatant and cell extract with ddH_2_O, nucleoside metabolites were determined using a high-performance liquid chromatography (HPLC) system (Agilent, USA) with an SB-AQ column (4.6 × 250 mm, 5 μm, Agilent) at 33 °C. Mobile phase A was 100% (v/v) methanol, whereas mobile phase B consisted of 0.5% (W/V) KH_2_PO_4_ at a pH of 4.5. HPLC was performed using a ratio of 90% A and 10% B at 1 mL/min with a monitor at 360 nm. All measurements were performed at least in triplicate and standard deviations (SD) were calculated from three independent experiments.

## Supplementary Information

**Additional file 1: Table S1**. Bacterial strains and plasmids used in this study. **Table S2**. Primers used in this study. **Table S3**. The properties of iBsu1103V2 model. **Table S4**. GDLS predicted knockout targets. **Table S5**. The specific reactions catalyzed by the enzymes encoded by *drm *and *fbaA*. **Table S6**. The metabolic fluxes of biomass and IMP by different methods. **Figure S1**. The metabolic pathway for purine synthesis. **Figure S2**. Inosine and hypoxanthine accumulation of engineered strains PN05, PN05-s0, PN05-s1 and PN05-s2. **Figure S3**. Cell growth of strains PN14, PN15 and PN16 during shake-flask cultivation. **Figure S4**. Cell growth of engineered strains PN16-p and PN18. **Figure S5**. Cell growth of engineered strains PN18, PN19 and PN20. **Figure S6**. Cell growth of engineered strain PN20 with different concentrations of xylose. **Figure S7**. Cell growth, residual glucose and inosine production of engineered strain PN20 in a 5-L fermenter. **Figure S8**. Cell growth, residual glucose and inosine production of engineered strain PN20 in minimum medium (MM).

**Additional file 2: Table S1**. The production yield, productivity, and conversion rate of inosine engineered strains. **Table S2**. Genes and reactions used in the iBsu1103V2 model. **Table S3**. The lower bounds and upper bounds of reactions based on the ^13^C flux data. **Table S4**. The targets possible targets for overproduction of IMP and inosine predicted by OptForce_MUST_.

## Data Availability

Data supporting the results of the article are included within this manuscript.

## Abbreviations

IMP: Inosine monophosphate
EMP: Glycolysis pathway/Embden–Meyerhof–Parnas pathway
PPP: Pentose phosphate pathway
PRPP: Phosphoribosyl pyrophosphate
AMP: Adenosine 5′-monophosphate
XMP: Xanthosine 5′-monophosphate
GMP: Guanosine 5′-monophosphate
GEM: Genome-scale metabolic network models
FBA: Flux balance analysis
GDLS: Genetic design through local search
ROOM: Minimum switch adjustment algorithm
PNP: Purine nucleoside phosphorylases
ORF: Opening reading frame
RBS: Ribosome binding site
*B. subtilis*: *Bacillus subtilis*
HPLC: High-performance liquid chromatography
OD_600_: Optical density at wavelength (λ) 600 nm
WT: Wild type
BS: *Bacillus subtilis*
SOE: Splice overlap extension
SD: Standard deviations
qRT-PCR: Real-time quantitative reverse transcription PCR
FM: Fermentation medium
MM: Minimum medium
