# Supplementary material for: In silico-guided metabolic engineering of Bacillus subtilis for efficient biosynthesis of purine nucleosides by blocking the key backflow nodes
Source: Biotechnol Biofuels Bioprod. 2022 Aug 11;15:82. doi: 10.1186/s13068-022-02179-x (PMC9367096; doi:10.1186/s13068-022-02179-x)
Supplement: Supplementary file 1 — Additional file 1: Table S1. Bacterial strains and plasmids used in this study. Table S2. Primers used in this study. Table S3. The properties of iBsu1103V2 model. Table S4. GDLS predicted knockout targets. Table S5. The specific reactions catalyzed by the enzymes encoded by drm and fbaA. Table S6. The metabolic fluxes of biomass and IMP by different methods. Figure S1. The metabolic pathway for purine synthesis. Figure S2. Inosine and hypoxanthine accumulation of engineered strains PN05, PN05-s0, PN05-s1 and PN05-s2. Figure S3. Cell growth of strains PN14, PN15 and PN16 during shake-flask cultivation. Figure S4. Cell growth of engineered strains PN16-p and PN18. Figure S5. Cell growth of engineered strains PN18, PN19 and PN20. Figure S6. Cell growth of engineered strain PN20 with different concentrations of xylose. Figure S7. Cell growth, residual glucose and inosine production of engineered strain PN20 in a 5-L fermenter. Figure S8. Cell growth, residual glucose and inosine production of engineered strain PN20 in minimum medium (MM). [file 13068_2022_2179_MOESM1_ESM.doc]

**Supporting Information**

*In silico*-guided metabolic engineering of *Bacillus subtilis* for efficient biosynthesis of purine nucleosides by blocking the key backflow nodes

Aihua Deng^1^, Qidi Qiu^1^, Qinyun Sun^1^, Zhenxiang Chen^1^, Junyue Wang^1,2^, Yu Zhang^1^, Shuwen Liu^1^, Tingyi Wen^1,3,4^*

^1^CAS Key Laboratory of Pathogenic Microbiology and Immunology, Institute of Microbiology, Chinese Academy of Sciences, Beijing 100101, China

^2^ College of Life Sciences, University of Chinese Academy of Sciences, Beijing 100049, China

^3^Savaid medical school, University of Chinese Academy of Sciences, Beijing, 100049, China

^4^China Innovation Academy for Green Manufacture, Chinese Academy of Sciences, Beijing, 100049, China

*Correspondence should be addressed to T.W. (wenty@im.ac.cn)

**Table S1 Bacterial strains and plasmids used in this study.**

| **Strains or plasmids** | **Relevant characteristics** | **References or sources** |
| --- | --- | --- |
| ***E. coli*** |  |  |
| EC135 | TOP10 Δ*dcm*::*FRT recA^+^*Δ*dam*::*FRT,* genotype: *F^-^ mcrA Δ(mrr-hsdRMS-mcrBC) φ80 lacZ ΔM15 ΔlacX74 nupG recA1 araD139 Δ(ara-leu)7697 galE15 galK16 rpsL(Str^R^) endA1 λ^-^* Δ*dcm*::*FRT recA^+^* Δ*dam*::*FRT* | [*^1^*](#_ENREF_1) |
| ***B. subtilis*** |  |  |
| W168 | Prototroph | BGSC |
| PN01 | W168 *ΔpurA* | This study |
| PN02 | W168 *ΔpurA ΔpupG* | This study |
| PN03 | W168 *ΔpurA ΔdeoD* | This study |
| PN04 | W168 *ΔpurA ΔpupG ΔdeoD* | This study |
| PN05 | W168 *ΔpurA ΔP_drm_* | This study |
| PN06 | W168 *ΔpurA drm** | This study |
| PN07 | W168 *ΔpurA Δdrm* | This study |
| PN05-S0 | W168 *ΔpurA ΔP_drm_* *lacA::P_xyl_* | This study |
| PN05-S1 | W168 *ΔpurA ΔP_drm_* *lacA::P_xyl_-drm* | This study |
| PN05-S2 | W168 *ΔpurA ΔP_drm_* *lacA::P_xyl_-pupG* | This study |
| PN08 | W168 *P_pur_*::*P_43_* | This study |
| PN09 | W168 *P_pur_*::*P_veg_* | This study |
| PN10 | W168 *P_pur_*::*P_ctc_* | This study |
| PN11 | W168 *P_pur_*::*P_gsiB_* | This study |
| PN12 | W168 *ΔpurA P_pur_::P_veg_* | This study |
| PN13 | W168 *ΔpurA ΔpupG P_pur_::P_veg_* | This study |
| PN14 | W168 *ΔpurA Δdrm P_pur_::P_veg_* | This study |
| PN15 | W168 ∆*purA* ∆*drm P_pur_::P_veg_* ∆*ywjH* | This study |
| PN16 | W168 ∆*purA* ∆*drm* P*_pur_*::P*_veg_* ∆*ywjH* ::*zwf* | This study |
| PN17 | W168 ∆*purA* ∆*drm* P*_pur_*::P*_veg_* ∆*ywjH*::*zwf /*pHT01-*scaf* | This study |
| PN18 | W168 ∆*purA* ∆*drm* P*_pur_*::P*_veg_* ∆*ywjH*::*zwf /*pHT01-*scaf-glcK-zwf-ykgB* | This study |
| PN19 | W168 ∆*purA* ∆*drm* P*_pur_*::P*_veg_* ∆*ywjH* ∆*pgi* ::*zwf* | This study |
| PN20 | W168 ∆*purA* ∆*drm* P*_pur_*::P*_veg_* ∆*ywjH*::*zwf* P*_pgi_*::P*_xly_* | This study |
| PN21 | PN20/pMK4-*purA* | This study |
| **Plasmids** |  |  |
| pWYE486 | A vector used for scarless genetic manipulation in *B. subtilis* | [*^2^*](#_ENREF_2) |
| pAX01 | *B. subtilis* integrative vector, xylose-inducible promoter | BGSC |
| pHT01 | *E. coli-Bacillus* shuttle plasmid, rolling circle replicative | Biovector |
| pMK4 | *B. subtilis* expression vector, *P_43_* promoter | BGSC |
| pWYE500 | pWYE486-*deoDUD* | This study |
| pWYE2838 | pWYE486-*drm-pupGUD* | This study |
| pWYE2840 | pWYE486-*purAUD* | This study |
| pWYE2846 | pWYE486-*drmUD* | This study |
| pWYE2847 | pWYE486-*pupGUD* | This study |
| pWYE2848 | pAX01-*drm* | This study |
| pWYE2849 | pAX01-*pupG* | This study |
| pWYE2852 | pMK4-*purA* | This study |
| pWYE2856 | pWYE486-*drm*UD* | This study |
| pWYE2861 | pWYE486-*P_pur_::P_43_UD* | This study |
| pWYE2862 | pWYE486-*P_pur_::P_veg_UD* | This study |
| pWYE2863 | pWYE486-*P_pur_::P_ctc_UD* | This study |
| pWYE2864 | pWYE486-*P_pur_::P_gsiB_UD* | This study |
| pWYE2866 | pWYE486- *ywjHUD* | This study |
| pWYE2867 | pWYE486- *pgiUD* | This study |
| pWYE2868 | pWYE486-P*_43_-zwfUD* | This study |
| pWYE2869 | pWYE486-P*_xly_-xylRUD* | This study |
| pWYE2870 | pHT01- *scaf^b^* | This study |
| pWYE2871 | pHT01- *scaf^b^-glcK-zwf-ykgB* | This study |

^a^ *Bacillus* Genetic Stock Center; *^b^* GBD-SH3-PDZ cassette; * Point mutation.

**Table 2** Primers used in this study.

| **Primer** | | **Sequences (5′-3′)** | **Description** | |
| --- | --- | --- | --- | --- |
| WB1036 | CTAGCTAGCGCTTCCTTTGCCTTCCTTTCCC | | | Knockout of *deoD* |
| WB1037 | GATGATAATTATATCAAGAGGCGTGCTGGGTG | | |
| WB1038 | CTCTTGATATAATTATCATCCAGCCCGTCCAT | | |
| WB1039 | CTAGCTAGCCTTTAGCCTCGGCTTGCTTCTG | | |
| WB1451 | GCGTGAGTGATGAAGGTTT | | | qRT-PCR of 16S rDNA |
| WB1452 | GCCGTGGCTTTCTGGTTA | | |
| WB1467 | CGGGGTACCGTCTTTTCAAGCAGCACA | | | Knockout of *purA* |
| WB1468 | TATTCGCGTCCGACCATGTCCGTGCACCTCCG | | |
| WB1469 | GGTGCACGGACATGGTCGGACGCGAATATGGA | | |
| WB1470 | ACATGCATGCATGGCTCTTAAAGATGTCG | | |
| WB1475 | CGGGGTACCGAGCGGTTATTGGACACG | | | Knockout of *drm-pupG* upstream |
| WB1476 | CTCCCAGCGTATCCCCGTTGACAATGTATTTG | | |
| WB1477 | ACATTGTCAACGGGGATACGCTGGGAGAAGAT | | |
| WB1478 | ACATGCATGCCCTTCAACAGTA | | |
| WB1539 | CTAGCTAGCGGCATTACTTCTTCACGC | | | Knockout of *pupG* |
| WB1540 | GGTTTGTTCCCATAGAAACAGTCCCCCTATTG | | |
| WB1541 | AGGGGGACTGTTTCTATGGGAACAAACCCGTT | | |
| WB1542 | CTAGCTAGCCCTCCATTTCATAATCCT | | |
| WB1535 | CTAGCTAGCGAGCGGTTATTGGACACG | | | Knockout of *drm* |
| WB1536 | ATTCTGTCCTTCAACTTGAAAGCCTCCTTTTT | | |
| WB1537 | AGGAGGCTTTCAAGTTGAAGGACAGAATTGAA | | |
| WB1538 | CTAGCTAGCTCCTGTCACAGCAGTGTA | | |
| WB1684 | GACCTTCTTACGAAACACC | | | qRT-PCR of *pupG* |
| WB1685 | TCGTGACTTAAAGGCTGAT | | |
| WB1686 | AGAAAGAGGCATCAAGGTG | | | qRT-PCR of *purE* |
| WB1687 | TTGGACGATGGAAAGAAG | | |
| WB1768 | TCTTTTCCAGCTATTGAAGCAATTCATCA | | | *drm* nonsense mutation |
| WB1769 | GAATTGCTTCAATAGCTGGAAAAGAGATC | | |
| WB1964 | CCCCACCTTCTAATAATGGTACCGCTATCACTTT | | | *P_43_* replacement |
| WB1965 | ATAGCGGTACCATTATTAGAAGGTGGGGAACAGA | | |
| WB1966 | CCACTTAATGGGTGTTCC | | | qRT-PCR of *prs* |
| WB1967 | TAGTCGGTCAGCCAGTTT | | |  |
| WB1968 | CCAAGGGAGCATCTTTCA | | | qRT-PCR of *purF* |
| WB1969 | GCGCCTTTCAGCATGGAT | | |
| WB1970 | TGCAGGTTTTCAACTGTTAGATCAATTTCCCTTC | | | *P_veg_* replacement |
| WB1971 | GGAAATTGATCTAACAGTTGAAAACCTGCATAGG | | |
| WB1972 | TGTTCCCCACCTTCTAATACTACATTTATTGTAC | | |
| WB1973 | TACAATAAATGTAGTATTAGAAGGTGGGGAACAG | | |
| WB1974 | TTTAATTGTTGAATTATTAGATCAATTTCCCTTC | | | *P_ctc_* replacement |
| WB1975 | GGAAATTGATCTAATAATTCAACAATTAAAGAGC | | |
| WB1976 | GTTCCCCACCTTCTAATGTCGTTTTAGTTGTCCT | | |
| WB1977 | GACAACTAAAACGACATTAGAAGGTGGGGAACAG | | |
| WB1978 | GTCCGTCTGCTTTCTGTTAGATCAATTTCCCTTC | | | *P_gsiB_* replacement |
| WB1979 | AGGGAAATTGATCTAACAGAAAGCAGACGGACAC | | |
| WB1980 | TTCCCCACCTTCTAATAATTGGTGTTGGTTGTTG | | |
| WB1981 | CAACCAACACCAATTATTAGAAGGTGGGGAACAG | | |
| WB3469 | CGGGGTACCAGCTTGCTCATTTCCACGGA | | | Knockout of *ywjH* |
| WB3443 | TTTGCCGCCCCTTTCAAAGCCTCCCTGATTAAGAA | | |
| WB3444 | CTTAATCAGGGAGGCTTTGAAAGGGGCGGCAAACAGCTT | | |
| WB3470 | ACATGCATGCAACCATTTCCCCATTCTCAA | | |
| WB3388 | TCCAATGACCGCCTGCTTGA | | | Identification of *ywjH* |
| WB3446 | GCTATGGTAAAAGCACTTATGG | | |
| WB3803 | ACATGCATGCCAGACGGTCATCGAAACAGCTC | | | Knockout of *pgi* |
| WB3804 | CCAGTCAGCTTTCTCACATGCTTGTCCCTCCATAACGG | | |
| WB3805 | CGTTATGGAGGGACAAGCATGTGAGAAAGCTGACTGGCAT | | |
| WB3806 | CGGGGTACCAGTAGCCATGATCGTATTCC | | |
| WB3807 | GTATACGGCATGGTTGACAT | | | Identification of *pgi* |
| WB3808 | GTTCATCGAGACTGCCCTGTA | | |
| WB4051 | ACATGCATGCGTGCACCAGGCCCTGTAGATATG | | | Integration of *zwf* |
| WB4052 | GCTGAGCTCTACAAGGAAGCCGCAAAATCAATTTCATTTACACAA | | |
| WB4053 | TTGTGTAAATGAAATTGATTTTGCGGCTTCCTTGTAGAGCTCAGC | | |
| WB4054 | GCTTTTGGTTGTTGGTTTGTTTTCACGTGTACATTCCTCTCTTACCTATAA | | |
| WB4055 | TTATAGGTAAGAGAGGAATGTACACGTGAAAACAAACCAACAACCAAAAGC | | |
| WB4056 | TTAAATCTTAACCTGAGCACAACAATTATATGTTCCACCAGTGTAAGCCG | | |
| WB4057 | CGGCTTACACTGGTGGAACATATAATTGTTGTGCT CAGGTTAAGATTTAA | | |
| WB4058 | ACATGCATGCCGAATAAGCGTTTGTTGGCATCC *Sph*1 | | |
| WB4059 | CACGATTACTGTCACAATTGC | | | Identification of *zwf* |
| WB4060 | CTCTAAGGATACGAGAATGAC | | |
| WB3890 | ACATGCATGCCAGACGGTCATCGAAACAGCTC | | | Construction of pMK4-*purA* |
| WB3891 | CCAGTCAGCTTTCTCACATGCTTGTCCCTCCATAACGG | | |
| WB3892 | CGTTATGGAGGGACAAGCATGTGAGAAAGCTGACTGGCAT | | |
| WB3893 | CGGGGTACCAGTAGCCATGATCGTATTCC | | |
| WB3894 | CAGTGAGCGCAACGCAATT | | | Identification of pMK4-*purA* |
| WB3895 | TCAGGCTGCGCAACTGTTG | | |
| WB3578 | TGAGCGGATAACAATTCCCAATTAAAGGAGGAAGGATCATGACCAAGGCAGATATTGG | | | Expression of *Scaf* |
| WB3579 | TAGGCGGGCTGCCCCGGGGACGTCGACTCTAGACTGCAGT TACTTGAAAT AAGGTGAGA | | |
| WB3580 | GGTTGTGTTGGAGGTTAAGTACAT | | | Expression of *zwf* |
| WB3583 | AAGATCGATGCCAGCGAACCAGA | | |  |
| WB3584 | ATGGATGAAATCTGGTTCGCTG | | | Expression of *glcK* |
| WB3585 | ATCCAGATCCTGAGCCAACAAGA | | |  |
| WB3586 | ATGGGCGTTAAAGAATCTCTTG | | | Expression of *ykgB* |
| WB3587 | TGACGTGAAAAAAGCCCGCTCATTA | | |  |
| WB3899 | CGGGGTACCGAAAACACGCCGCTTCAGGATAG | | | P*_pgi_* replaced by P*_xly_-xlyR* |
| WB3907 | CCCTATAAGTTAGGAGCTCAAGAAAGGGCGGAATGACTGG | | |
| WB3902 | GTCAAAGCGTACATGCGTCATGGATCCCATTTCCCCCTTTG | | |
| WB3903 | CAAAGGGGGAAATGGGATCCATGACGCATGTACGCTTTGAC | | |
| WB3904 | ACATGCATGCCGCGCTCTTTATCAGTTGTTG | | |
| WB3908 | CCAGTCATTCCGCCCTTTCTTGAGCTCCTAACTTATAGGG | | |
| WB3905 | GATGAGCCACGTATTCGAAC | | | Identification of P*_pgi_* replaced by P*_xly_-xlyR* |
| WB3906 | ATTGGTATGCTGGGTTATCTTC | | |

**Table S3** The properties of *i*Bsu1103V2 model [*^3^*](#_ENREF_3).

| ***i*Bsu1103V2** | **Number** |
| --- | --- |
| Reactions | 1451 |
| Genes | 1103 |
| Exchange reactions | 249 |
| Metabolites | 1156 |

**Table S4** GDLS predicted knockout targets.

| **Strategies** | **Reaction ID of knockouts** | **Biomass Flux**_max_ (h^-1^) | **rxn00832 Flux**_max_ (mmol/gDW/h) | **Enzyme** | **Gene** |
| --- | --- | --- | --- | --- | --- |
| 1 | rxn01986 | 0.243 | 0.293 | phosphopentomutase | *drm* |
|  | rxn01333 |  |  | transaldolase | *ywjH* |
| 2 | rxn01986 | 0.243 | 0.293 | phosphopentomutase | *drm* |
|  | rxn00786 |  |  | fructose-bisphosphate aldolase | *fbaA* |

**Table S5** The specific reactions catalyzed by the enzymes encoded by the *drm* and *fbaA*.

| **Gene** | **Enzyme** | **Reaction ID** | **Reaction Equation** |
| --- | --- | --- | --- |
| *drm* | phosphopentomutase | rxn01986 | cpd00509 <=> cpd00510 |
|  |  | rxn00778 | cpd00475 <=> cpd00101 |
|  | fructose-bisphosphate aldolase | rxn00786 | cpd00290 <=> cpd00102 + cpd00095 |
| *fbaA* |  | rxn01334 | cpd00349 <=> cpd00236 + cpd00095 |
|  |  | rxn01870 | cpd00802 <=> cpd00095 + cpd00448 |

**Table S6** The metabolic fluxes of biomass and IMP by different methods.

| **Methods** | **Mutant metabolic fluxes** |  |
| --- | --- | --- |
|  | **Specific growth rate/ Biomass** (h^-1^) | **IMP/Inosine synthesis rate** (mmol/gDW/h) |
| FBA_max biomass_ | 0.263 | 0.070 |
| FBA_max IMP_ | 0.000 | 1.450 |
| GDLS_max_ | 0.243 | 0.293 |
| ROOM_min_ | 0.243 | 0.083 |
| ^a^Experiment_MM_ | 0.011 | 0.220 |
| ^b^Experiment_FM_ | 0.172-0.237 | 0.081-0.096 |

^a^ The shake-flask experiment using minimum medium (MM).

^b^ The shake-flask and fed-batch experiments using fermentation medium (FM).

**
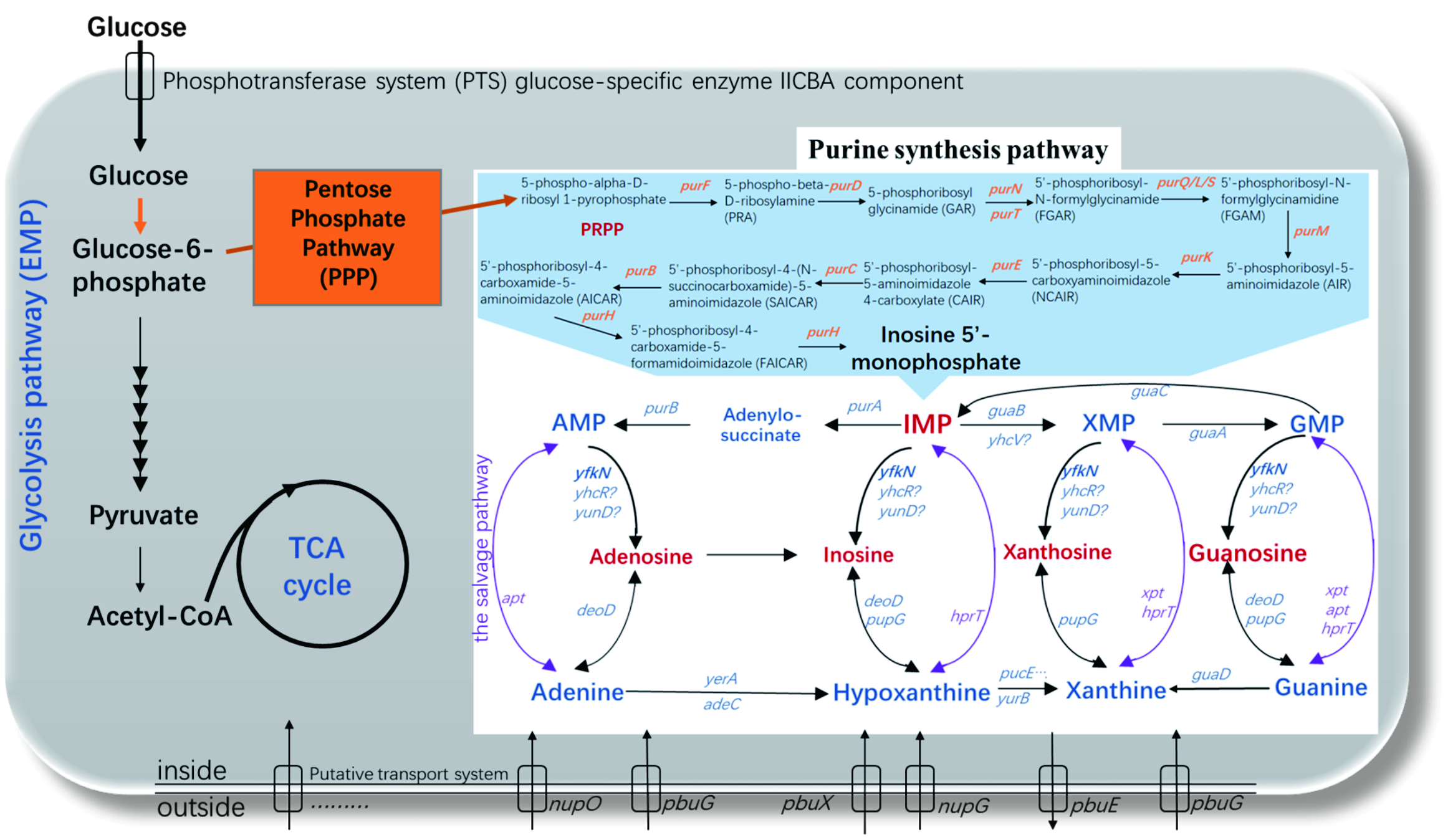
**

**Figure S1. The metabolic pathway for purine synthesis.**

**
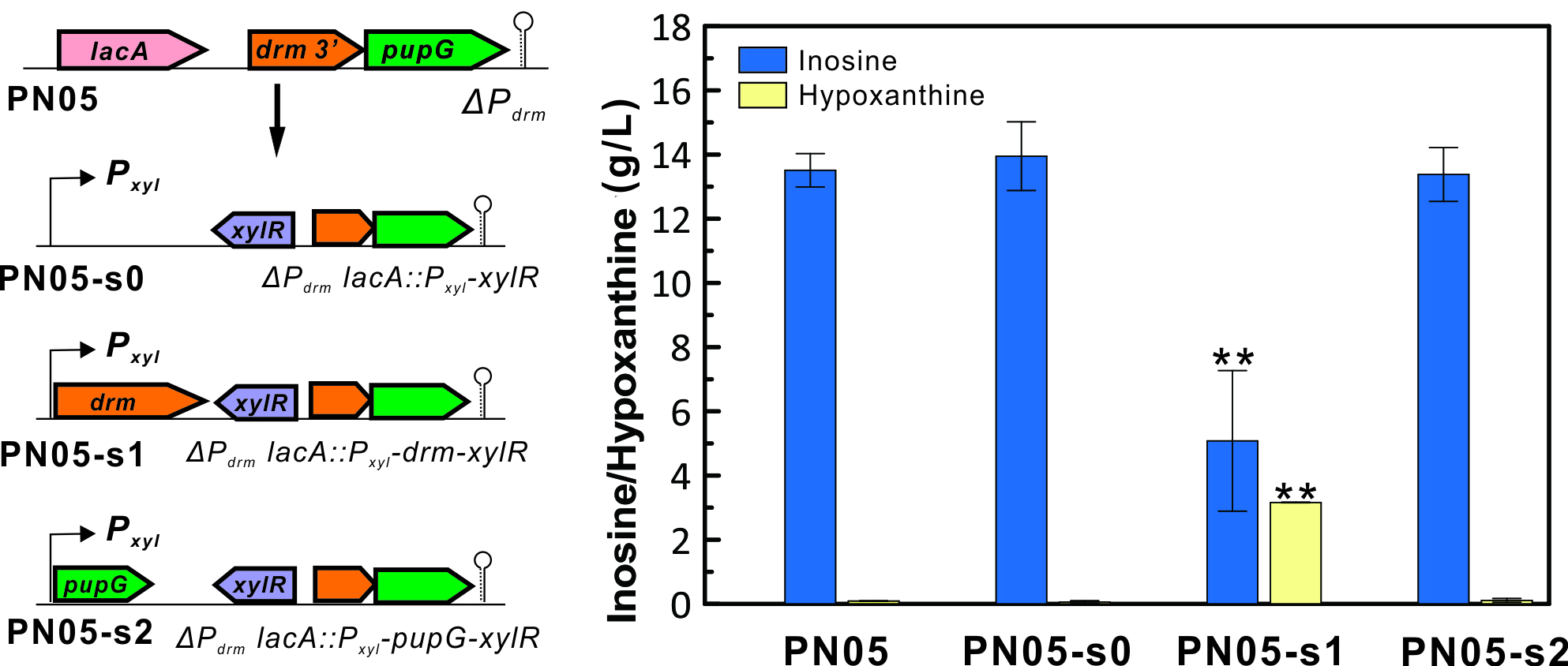
**

**Figure S2. Inosine and hypoxanthine accumulation of engineered strains PN05, PN05-s0, PN05-s1 and PN05-s2.** The *P_xyl_*-controlled gene *drm* or *pupG* was complemented in the *lacA* site of PN05 (PN01 *ΔP_drm_*) to generate PN05-s1 (PN01 *ΔP_drm_ lacA::P_xyl_ -drm-xylR*) or PN05-s2 (PN01 *ΔP_drm_ lacA::P_xyl_ -pupG -xylR*). The strain PN05-s0 (PN01 *ΔP_drm_ lacA::P_xyl_ -xylR*) integrating the promoter *P_xyl_* and repressor gene *xylR* was used as the control. Data were shown in mean values from three biological replicates and the standard deviations were presented. All error bars indicate ± SD, *n* = 3. A value of *P* less than 0.05 was regarded to be a significant difference using the T-test (**, *P* < 0.01).


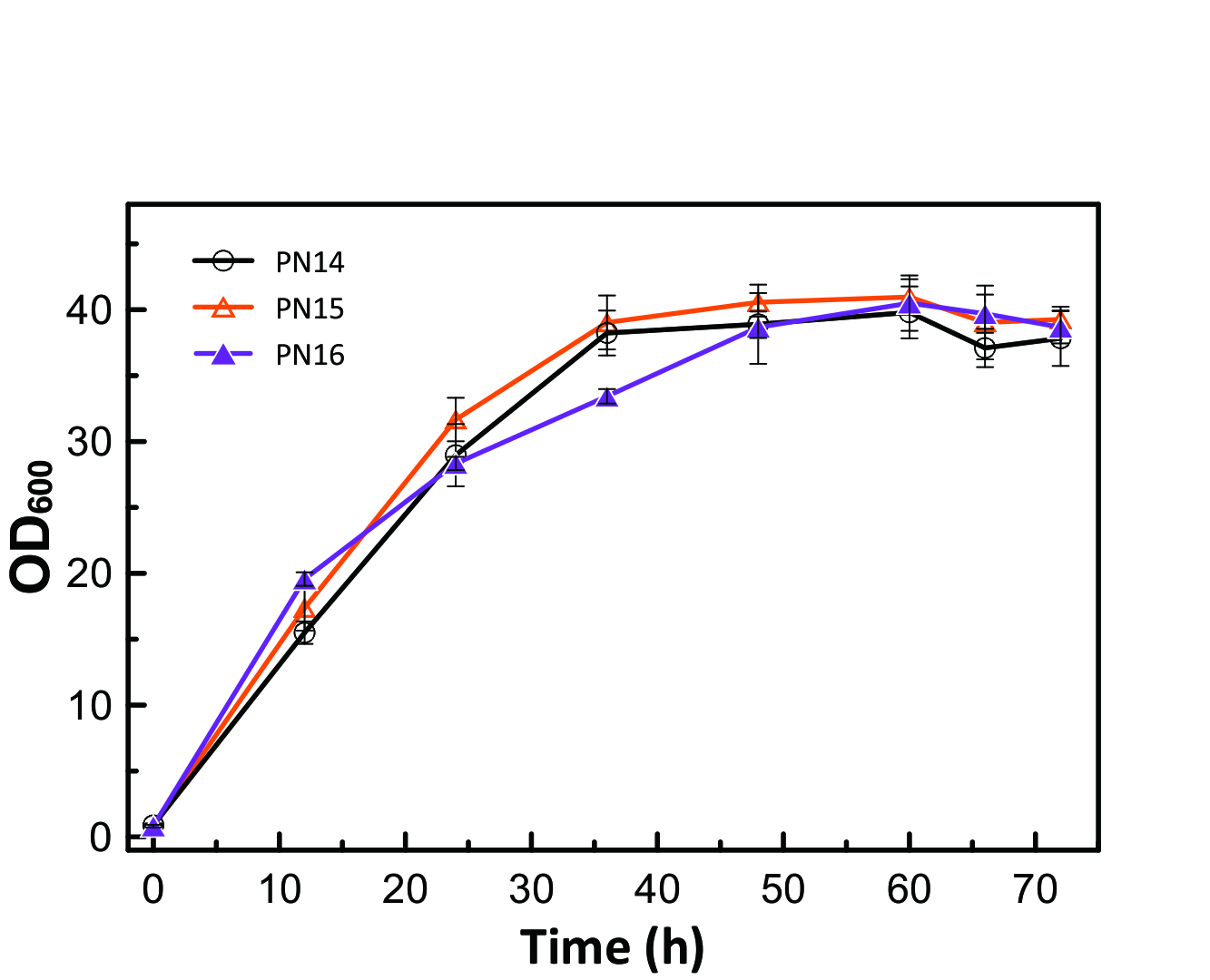


**Figure S3. Cell growth of strains PN14, PN15 and PN16 during shake-flask cultivation.** Data shown are mean values from three biological replicates and the standard deviations were presented.

**
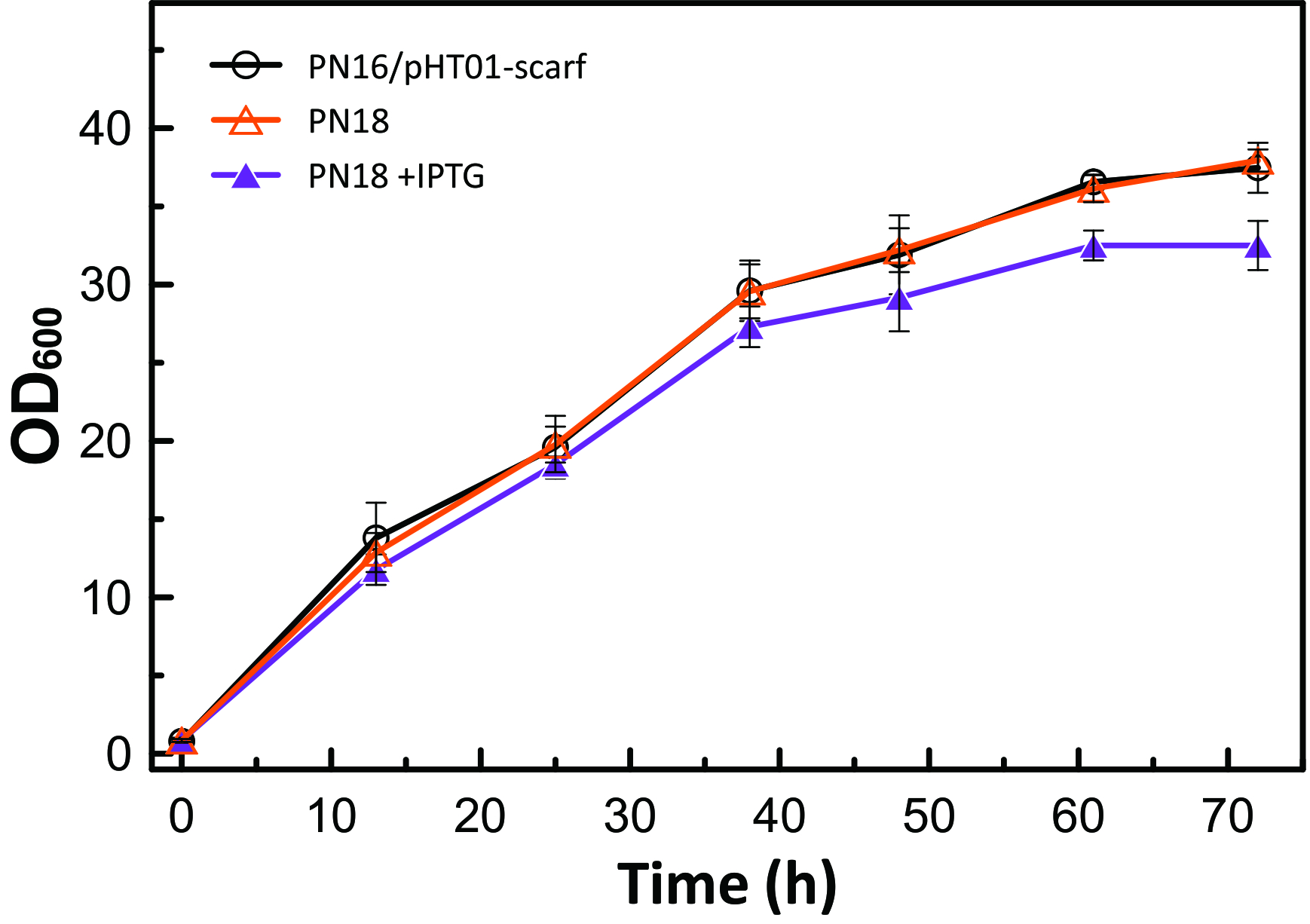
**

**Figure S4. Cell growth of engineered strains PN16-p and PN18.** The data shown are mean values from three biological replicates. All error bars indicate ± SD, *n* = 3.

**
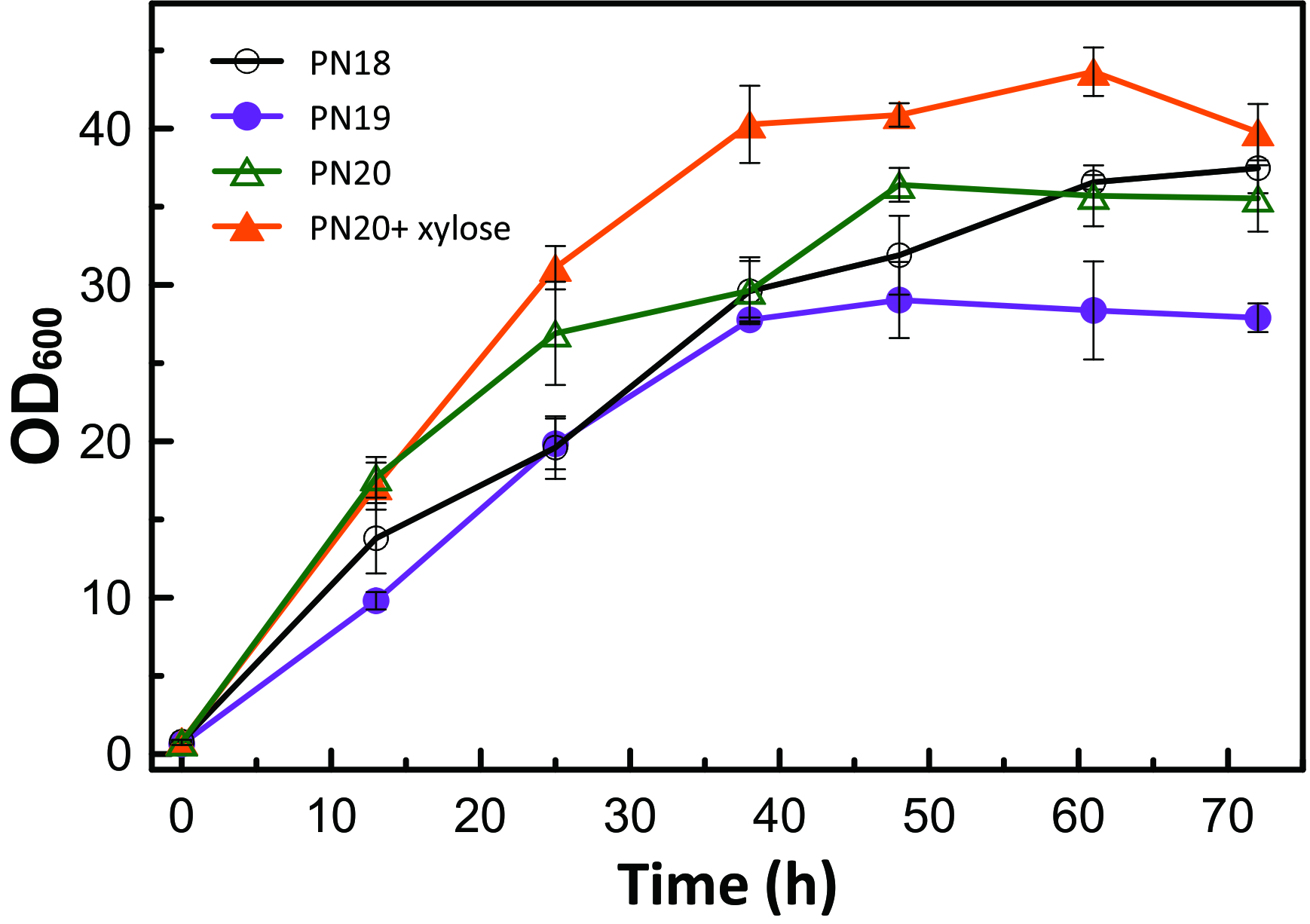
**

**Figure S5. Cell growth of engineered strains PN18, PN19 and PN20.** The data shown are mean values from three biological replicates. All error bars indicate ± SD, *n* = 3.


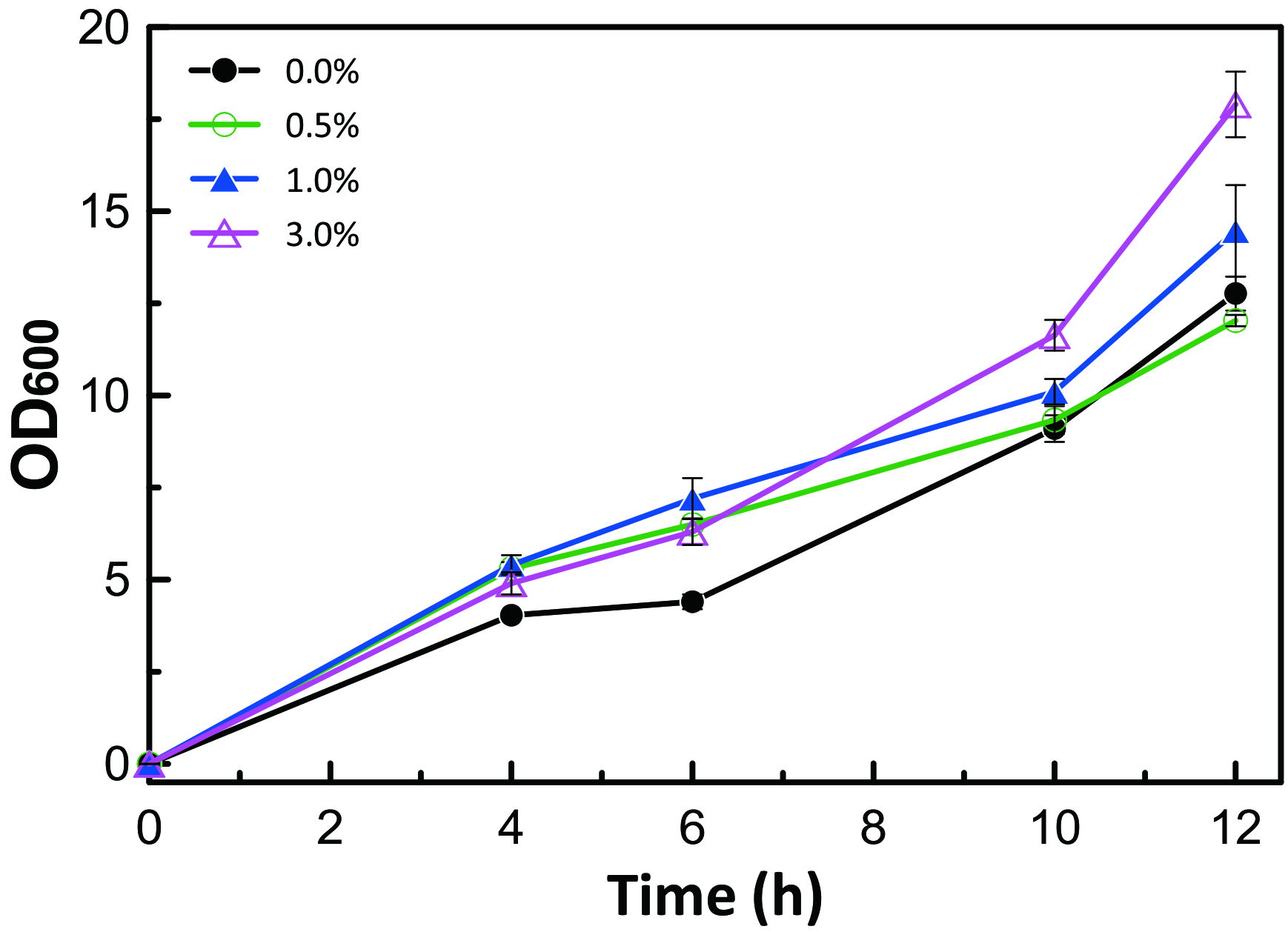


**Figure S6. Cell growth of engineered strain PN20 with different concentrations of xylose.** The data shown are mean values from three biological replicates. All error bars indicate ± SD, *n* = 3.

**
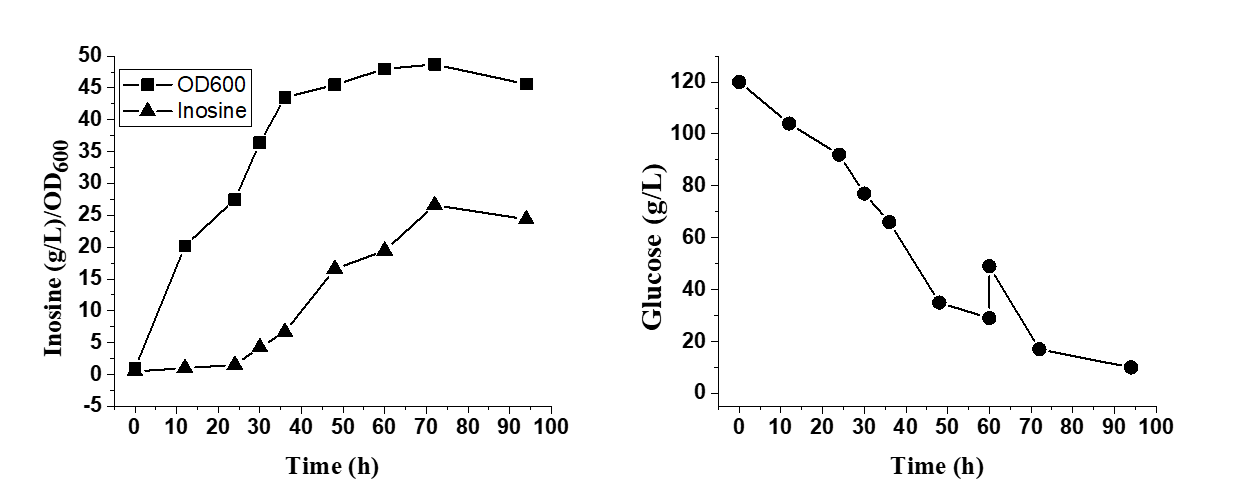
**

**Figure S7. Cell growth, residual glucose and inosine production of engineered strain PN20 in a 5-L fermenter.** A 2 L of fermentation medium (FM) were used for fed-batch culture in a 5-L fermenter (Shanghai bailun biological technology co., LTD). The seed medium and fermentation medium were the same as described as that of shake-flask cultivation. The temperature was maintained at 36°C and pH was maintained at 7.0 by the addition of ammonia. Dissolved oxygen tension was maintained at 30% of air saturation.


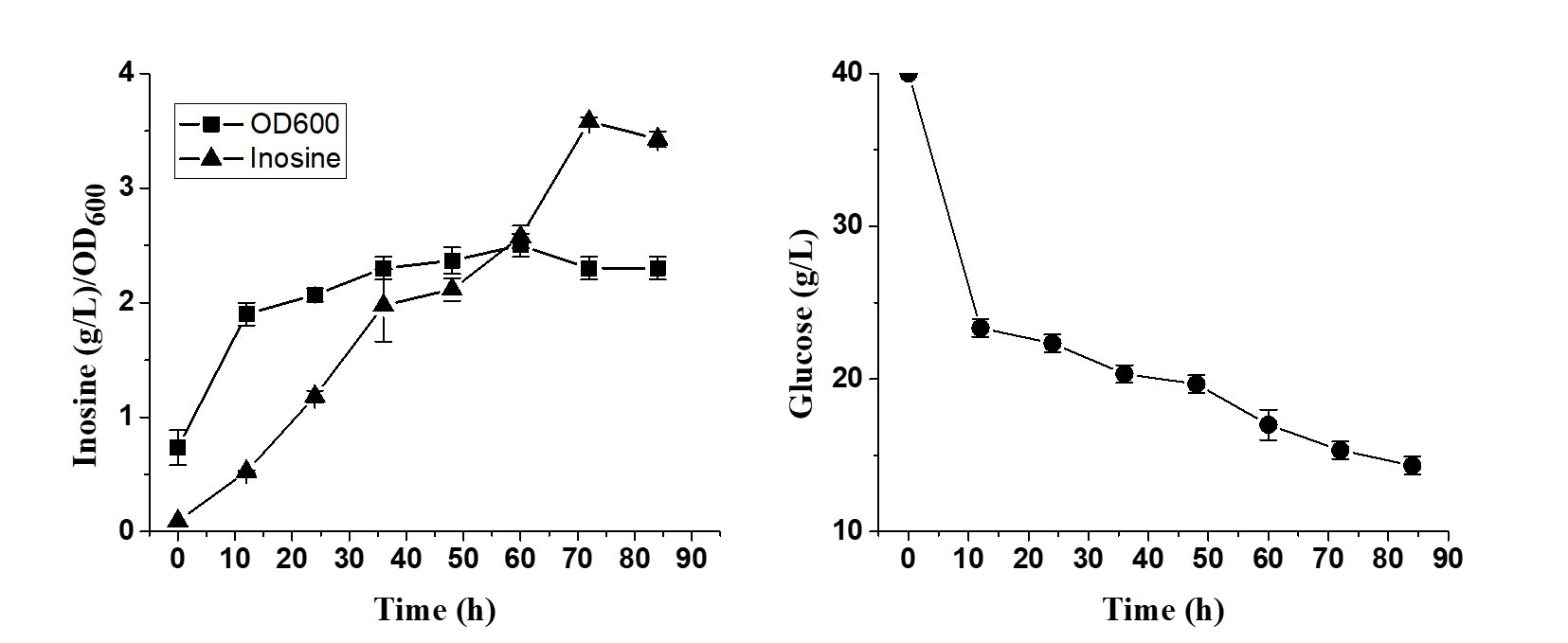


**Figure S8. Cell growth, residual glucose and inosine production of engineered strain PN20 in minimum medium (MM).** MM consists 40.00 g/L glucose, 20.00 g/L ammonium sulfate, 0.50 g/L K_2_HPO_4_, 0.50 g/L KH_2_PO_4_, 0.40 g/L MgSO_4_, and 5 mL/L trace metal elements (6.00 g/L FeSO_4_·7H_2_O, 1.35 g/L CaCl_2_, 0.8 g/L ZnSO_4_·7H_2_O, 1.5 g/L MnSO_4_·4H_2_O, 0.15 g/L CuSO_4_·5 H_2_O, 0.20 g/L (NH_4_)_6_Mo_7_O_24_·4H_2_O, 0.10 g/L H_3_BO_3_, 0.25 g/L CoCl_2_·6H_2_O and 10 mL/L 35% HCl). Considering PurA deficiency of the engineered strain, 0.3 g/L adenine was supplemented in MM. The data shown are mean values from three biological replicates. All error bars indicate ± SD, *n* = 3.

**References**

[1] Zhang, G. Q., Wang, W. Z., Deng, A. H., Sun, Z. P., Zhang, Y., Liang, Y., Che, Y. S., and Wen, T. Y. (2012) A Mimicking-of-DNA-Methylation-Patterns pipeline for overcoming the restriction barrier of bacteria, *PLoS Genet.* *8*, e1002987.

[2] Wu, J., Deng, A., Sun, Q., Bai, H., Sun, Z., Shang, X., Zhang, Y., Liu, Q., Liang, Y., Liu, S., Che, Y., and Wen, T. (2018) Bacterial genome editing via a designed toxin-antitoxin cassette, *ACS Synth. Biol.* *7*, 822-831.

[3] Tanaka, K., Henry, C. S., Zinner, J. F., Jolivet, E., Cohoon, M. P., Xia, F. F., Bidnenko, V., Ehrlich, S. D., Stevens, R. L., and Noirot, P. (2013) Building the repertoire of dispensable chromosome regions in *Bacillus subtilis* entails major refinement of cognate large-scale metabolic model, *Nucleic Acids Res.* *41*, 687-699.
